# Controllable CO_2_ electrocatalytic reduction via ferroelectric switching on single atom anchored In_2_Se_3_ monolayer

**DOI:** 10.1038/s41467-021-25426-5

**Published:** 2021-08-26

**Authors:** Lin Ju, Xin Tan, Xin Mao, Yuantong Gu, Sean Smith, Aijun Du, Zhongfang Chen, Changfeng Chen, Liangzhi Kou

**Affiliations:** 1 School of Mechanical, Medical and Process Engineering Faculty, Queensland University of Technology, Brisbane, QLD Australia; 2 School of Physics and Electric Engineering, Anyang Normal University, Anyang, China; 3 Integrated Materials Design Laboratory, Department of Applied Mathematics, Research School of Physics, The Australian National University, Canberra, Australian Captial Territory Australia; 4 School of Chemistry and Physics, Queensland University of Technology, Brisbane, QLD Australia; 5 Center for Materials Science, Queensland University of Technology, Brisbane, QLD Australia; 6 Centre for Biomedical Technologies, Queensland University of Technology, Brisbane, QLD Australia; 7 Department of Chemistry, University of Puerto Rico, Rio Piedras Campus, San Juan, PR USA; 8 Department of Physics and Astronomy, University of Nevada, Las Vegas, NV USA

**Keywords:** Electrocatalysis, Computational chemistry, Electrocatalysis

## Abstract

Efficient and selective CO_2_ electroreduction into chemical fuels promises to alleviate environmental pollution and energy crisis, but it relies on catalysts with controllable product selectivity and reaction path. Here, by means of first-principles calculations, we identify six ferroelectric catalysts comprising transition-metal atoms anchored on In_2_Se_3_ monolayer, whose catalytic performance can be controlled by ferroelectric switching based on adjusted *d*-band center and occupation of supported metal atoms. The polarization dependent activation allows effective control of the limiting potential of CO_2_ reduction on TM@In_2_Se_3_ (TM = Ni, Pd, Rh, Nb, and Re) as well as the reaction paths and final products on Nb@In_2_Se_3_ and Re@In_2_Se_3_. Interestingly, the ferroelectric switching can even reactivate the stuck catalytic CO_2_ reduction on Zr@In_2_Se_3_. The fairly low limiting potential and the unique ferroelectric controllable CO_2_ catalytic performance on atomically dispersed transition-metals on In_2_Se_3_ clearly distinguish them from traditional single atom catalysts, and open an avenue toward improving catalytic activity and selectivity for efficient and controllable electrochemical CO_2_ reduction reaction.

## Abstract (web summary)

Electroreduction of CO_2_ into chemical fuels holds promise for mitigating environmental pollution and energy crisis. This work presents a distinct design of ferroelectric catalysts with high catalytic activity and selectivity for efficient and controllable electrochemical CO_2_ reduction reaction.

## Introduction

The ever-rising global energy consumption and its negative impact on the environment are major challenges in front of humanbeing in the 21^st^ century^1–7^. Public health is severely threatened by the greenhouse effect due to the excessive fossil fuel usage. It is highly desirable to develop technologies that can convert the greenhouse gas (i.e., CO_2_) into sustainable and clean energy sources. Among various possibilities, catalytic reduction of CO_2_ into hydrocarbon fuels, which features environmental friendliness, high efficiency, and low cost, has been recognized as one of the most promising approaches. Single atom catalysts (SACs), first proposed for CO oxidation in 2011^8^, offer us excellent candidates to activate and convert CO_2_ efficiently when trasition metal (TM) atoms are used^9–13^, because the coexistence of empty and occupied TM *d*-orbitals can accept lone-pair electrons, and then back-donate these electrons to the antibonding orbitals to weaken the C=O bonds. Notably, two-dimensional (2D) materials serve as promising substrates for atomically dispersed transition metal atoms for CO_2_ reduction due to their large surface-to-volume ratios, short carrier diffusion distances, unique electronic properties, and abundant active sites. The catalytic performance of these 2D SACs stems from versatile advantageous characteristics. For example, catalysts with singly dispersed Ni, Co, Cu, Pt, and Pd atoms on graphene^14^, Pt or Pd atom anchored on C_3_N_4_^15^, V-β_12_ boron monolayer^16^ and Co decorated metal–organic frameworks^17,18^ all have shown great potential for CO_2_ reduction.

Despite latest advances, there is still plenty of room to improve the catalytic activity and selectivity of CO_2_ reduction in order to satisfy large-scale industrial applications. Recent studies have shown that electric polarization plays an important role in determining catalytic activities and selectivities, and can greatly improve the catalytic performance compared with the unpolarized counterparts^19–21^. For instance, Janus TM dichalcogenides possess better catalytic abilities for water splitting and N_2_ fixation^19–21^, since the catalysis-related properties, including surface stoichiometry, electronic structure^22–26^, adsorption strengths, and reaction activation energies, can be tuned by the polarization^27^; Moreover, by utilizing the polarization from the substrate, the overpotential of oxygen evolution reaction on TiO_2_ surface can be strongly reduced^28^.

Inspired by these recent achivements, we expect that ferroelectrics, *viz*., materials with switchable ferroelectric polarization, hold promise as efficient catalysts since their unique reversible polarization provides an additional mechanism to adjust empty and occupied *d-*orbitals of adsorbed metal atoms. The activation of reactants can thus be controlled, while the catalytic properties of the supported metal, such as activity^29–32^ and selectivity, can be tuned^33^. These expectations have been partially confirmed in ABO_3_ perovskite ferroelectrics. For instance, reversing the polarization of ferroelectric PbTiO_3_ substrate makes CrO_2_ monolayer overcome the limiting factor stemming from the Sabatier principle, and thus display excellent catalytic behaviours for both NO_x_ decomposition and CO oxidation^34^. Reorienting the polarization direction of the ferroelectric PbTiO_3_ substrate can dramatically change the chemisorption energies of CO, O, C, and N on the PbTiO_3_-supported Pt films, and alter the reaction paths of the dissociations^29^. The oxygen evolution reaction activity of TiO_2_ film on a ferroelectric SrTiO_3_ substrate can be strongly enhanced relative to unsupported TiO_2_ due to the presence of dynamic dipoles in response to the charge on the adsorbed species, while the reaction path can be modulated via ferroelectric switching^28^. However, these predictions have not been experimentally realized so far, mainly due to the instability and depolarization in traditional ABO_3_ perovskite ferroelectrics when the thickness is smaller than a critical value. Encouragingly, the emergence of 2D ferroelectric materials, exemplified by In_2_Se_3_^35^, CuInP_2_S_6_^36^, and SnTe^37^, provides an excellent opportunity to verify controllable catalysis in ferroelectrics. The realization of ferroelectricity at room temperature makes it feasible for 2D ferroelectrics to be used for controllable catalytic CO_2_ reduction.

In this work, based on the experimentally available 2D ferroelectrics, we theoretically investigate the potential of using transition metal decorated α-In_2_Se_3_ monolayers as the ferroelectric SACs toward electrochemical CO_2_ reduction. Our efforts identify six catalysts with singly dispersed TM atoms anchored on the 2D ferroelectric substrate, namely α-In_2_Se_3_ monolayer, with polarization downwards or upwards (denoted as TM@P ↓ -In_2_Se_3_ or TM@P ↑ -In_2_Se_3_). The switchable polarization can not only alter the reaction barrier and paths of CO_2_ reduction, but also lead to different final products. It can even reactivate the stuck CO_2_ reduction. These performance improvements stem from the synergistic effects of adjusted empty and occupied *d-*orbitals (*d* orbital center) of adsorbed metal atom, polarization dependent electron transfer, and CO_2_ adsorption energies under ferroelectric switching. These ferroelectric SACs, catalytic mechanism, and the polarization dependent catalytic CO_2_ reduction open an avenue for controllable CO_2_ reduction reaction (CO_2_RR), and introduce a feasible approach to significantly improve the efficiency of such reactions.

## Results

### Screening ferroelectric catalysts

**Fig. 1:**
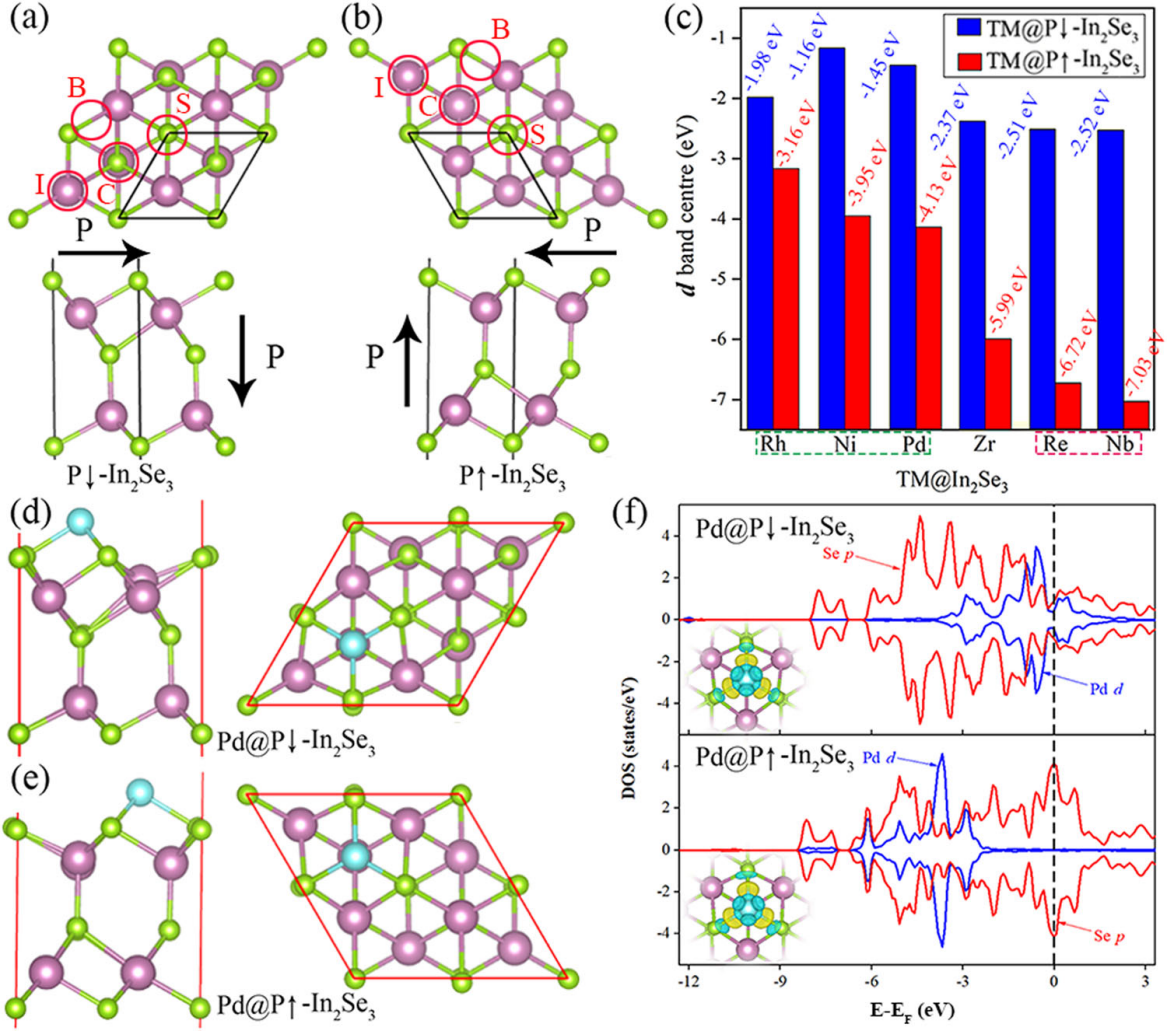
Geometries and electronic structures of the ferroelectric catalysts. Top and side views of the optimized α-In_2_Se_3_ monolayer with **a** downward (P ↓ ) and **b** upward (P ↑ ) polarization. Red circles denote selected adsorption sites: I, top of the In atom; C, center of the six-membered ring; B, top of the In-Se bond; S, top of the Se atom. The black rhombus represent the unitcell of In_2_Se_3_. **c** The *d* band center of TM@In_2_Se_3_ (TM = Ni, Pd, Rh, Zr, Nb and Re). Top and side views of the optimized configuration of **d** Pd@P ↓ -2×2 In_2_Se_3_ and **e** Pd@P ↑ -2×2 In_2_Se_3_, the supercell is indicated by the red rhombus. **f** The partial density of states of Pd@P ↓ -In_2_Se_3_ and Pd@P ↑ -In_2_Se_3_. The insets show the 3D differential charge density plots obtained with the model shown in Fig. 1d and e. The isosurfaces are 0.005 e/Å^3^. Charge accumulation and depletion are marked by the yellow and blue regions, respectively.

The ferroelectric α-In_2_Se_3_ monolayer was chosen as the substrate of potential catalysts due to the following reasons: (1) it is the ground-state phase among possible phases (see Supplementary Fig. 1), even in the presence of adatoms and in electrochemical environment (see Supplementary Fig. 2 and Supplementary Tables 1–2), which is consistent with the recent theoretical finding by Ding et al.^38^ Note that two α-In_2_Se_3_ (α and α’) phases have been predicted to share similar atomic structures and are almost energetically degenerate, and they have the same CO_2_RR performance (Supplementary Fig. 3). We thus mainly focus on α- In_2_Se_3_ in this study. (2) it is stable at room temperature and has been experimentally synthesized^35,39^, and its ferroelectricity with switchable polarization under room temperature has been explicitly demonstrated^35,40^. With the polarization locking from the asymmetric arrangement of its quintuple layers (see Fig. 1a, b), their orientations can be reversed by the shift of the middle Se layer^38^. However, as a ferroelectric semiconductor with a sizable band gap (1.46 eV)^38^, α-In_2_Se_3_ monolayer does not have enough electrons to be injected into the antibonding 2π_u_ orbitals of CO_2_ so that the strong *sp*-hybridization symmetry of the carbon atom can not be disrupted^41^. Thus, the material itself is not suitable as a catalyst for CO_2_ reduction^42^, which is corroborated by our theoretical study: upon adsorption on In_2_Se_3_, the inherent linear O=C=O structure of CO_2_ molecule is well maintained (see Supplementary Fig. 4 and Supplementary Table 3).

To activate electrochemical CO_2_ reduction, we introduced transition metal atoms to decorate α-In_2_Se_3_ monolayer, which provide extra electrons to break the strong *sp* hybridization, thus activating the CO_2_ molecules^29,42–44^. Compared with conventional metal catalysts, SACs normally exhibit higher catalytic performances because of the high ratio of low-coordinated configurations^8,45,46^. However, the transition metals have to be screened since only the SACs with well balanced empty/occupied *d* orbitals can exhibit optimal catalytic performance. To find suitable SACs, 29 transition metal atoms were chosen and chemically adsorbed (see Supplementary Fig. 5) on both surfaces of the In_2_Se_3_ monolayer, and the energetically most favorable configurations were identified. The possible metal substitutions into the In_2_Se_3_ monolayer were excluded due to the large formation energy of In vacancy, high diffusion barriers of In atoms (see Supplementary Fig. 6), and low CO_2_RR activities (see Supplementary Fig. 7). Then we evaluated all the composites based on two key criteria. First, to ensure the stability of the active site, the single transition metal atom should be steadily adsorbed on the In_2_Se_3_ monolayer surface without breaking the underlying structure, and the favourable adsorption site should not change significantly after the polarization switching; second, the single transition metal atom should be able to activate CO_2_, in other words, the linear structure of O=C=O should be broken after adsorption, at least in one polarization state.

After a comprehensive investigation of the energetically most favorable adsorption configurations (Fig. 1a, b) and their stabilities, we screened out six TM atoms (TM = Ni, Nb, Pd, Re, Rh and Zr) as promising catalysts toward CO_2_ reduction for further examinations. Other 23 candidates are either unstable or cannot effectively activate CO_2_ molecules (see details in the *Supplementary Information*).

### Stability of SACs and polarization dependent *d* band center

Although the screened TM@In_2_Se_3_ are stable (without structural breaking) and able to activate CO_2_ molecules, transition metal atoms need to uniformly disperse on the substrate to prevent any aggregation of the deposited atoms and maximize the catalytic efficiency^47^.

To achieve this goal, strong binding strength, high diffusion energy barrier, and negative clustering energy are essential^15,48–54^. At the energetically most favorable site, all the six TM atoms have rather strong binding energies (Table 1) that are comparable to previously predicated SACs^15,49,55,56^, indicating sufficiently strong interactions between the metal atoms and the substrate, as evidenced by the strong TM–Se bonds (Fig. 1d, e and Supplementary Fig. 8). To provide the reference for the binding strengths, we compared the binding energies of TM atoms in ferroelectric SACs with the ones in the corresponding molecular precursors, it is found that the adsorption of TM (e.g. Pd, Rh and Ni) atoms on In_2_Se_3_ is energetically preferred, indicating the feasibility of the SACs to be synthesized based on the corresponding molecular precursors (see Supplementary Table 4). The chemical bonding interaction is illustrated by the substantial partial charge accumulation between the TM atoms and the surrounding Se atoms (see Table 1, and Fig. 1f and Supplementary Fig. 8) as well as the strong hybridization of Se *p* and TM *d* orbitals (shown in Fig. 1f and Supplementary Fig. 9) that is similar to the case of TM atoms adsorbed on 2D carbo-nitride^15,49,55^. Moreover, the high diffusion barriers of TM atoms on the surfaces can effectively prevent the formation of metal clusters (see Supplementary Fig. 10), though specific values depend on the ferroelectric polarization.

**Table 1:** Parameters for the pure TM@In_2_Se_3_ and CO_2_ adsorbed TM@In_2_Se_3_: adsorption site (*S*_*ad*_), binding energies of TM atom (*E*_b-TM_ in eV/atom) and CO_2_ molecule (*E*_b-CO2_ in eV/molecule), charge lost from the adsorbed TM atoms (*Q*_TM_ in *e*/atom), average TM-Se bond length (*l*_TM-Se_ in Å), migration barrier (*E*_ba-TM_ in eV), charge gained by the adsorbed CO_2_ molecule (*Q*_CO2_ in *e*/molecule), and bond angle (∠OCO in °).

**Catalysts**	***S***_***ad***_	***E***_**b-TM**_	***E***_**ba-TM**_	***Q***_**TM**_	***l***_**TM-Se**_	***E***_**b-CO2**_	***Q***_**CO2**_	**∠OCO**
Ni@P ↓ -In_2_Se_3_	C	−2.72	1.78	0.40	2.31	−1.39	0.45	146.4
Ni@P ↑ -In_2_Se_3_	C	−1.97	1.41	0.37	2.37	−1.08	0.40	146.5
Pd@P ↓ -In_2_Se_3_	C	−1.69	1.19	0.14	2.50	−0.84	0.33	149.2
Pd@P ↑ -In_2_Se_3_	C	−1.14	0.84	0.12	2.58	−0.79	0.28	151.2
Rh@P ↓ -In_2_Se_3_	I	−4.09	4.19	0.12	2.38	−1.05	0.40	146.5
Rh@P ↑ -In_2_Se_3_	I	−2.36	2.49	0.08	2.49	−0.76	0.26	153.9
Zr@P ↓ -In_2_Se_3_	I	−6.26	6.13	2.10	2.58	−0.01	0.02	179.2
Zr@P ↑ -In_2_Se_3_	I	−5.01	5.06	2.03	2.61	−3.39	1.13	126.2
Nb@P ↓ -In_2_Se_3_	I	−7.08	4.63	1.70	2.46	−2.54	0.92	131.1
Nb@P ↑ -In_2_Se_3_	I	−4.73	4.21	1.61	2.59	−2.47	0.85	134.7
Re@P ↓ -In_2_Se_3_	C	−2.36	5.18	0.90	2.34	−2.11	0.69	134.8
Re@P ↑ -In_2_Se_3_	C	−1.07	3.25	0.71	2.36	−2.02	0.61	136.8

To further demonstrate the structural stability of the SACs chosen in this work, we also conducted clustering energy calculations and *ab* initio molecular dynamics (AIMD) simulations with a Nose–Hoover thermostat at 300 K on Pd@In_2_Se_3_ as a representative example. The neagive clustering energies (−0.3 eV and −0.04 eV for Pd@P ↓ -In_2_Se_3_ and Pd@P ↑ -In_2_Se_3_, respectively; see details in SI) indicate that it is not energetically favorable to form clusters on the catalyst surface. In the AIMD simulations, the out-of-plane polarization of ferroelectric In_2_Se_3_ monolayer can be well retained, and the metal atom stays anchored at the energentically favorable site even at room-temperature (300 K) for at least 15 ps (see Supplementary Fig. 11a). Besides, AIMD simulations at 300 K are also performed to investigate two dispersedly adsorbed Pd atoms on P ↓ -In_2_Se_3_ (2 Pd@P ↓ -In_2_Se_3_). The two Pd atoms can maintain dispersedly adsorbed features without metal clustering and structural phase transition for 15 ps (see Supplementary Fig. 11b). The distance between the two Pd atoms stays almost unchanged (as shown in the inset of Supplementary Fig. 11). The possible metal (e.g. Pd and Nb) agglomerations are excluded by the further kinetic Monte Carlo (kMC) simulations, the clusters will not form at the surface for 100 seconds at the room-temperature (see Supplementary Figs. 12–14). Overall, the strong adsorption energies, high diffusion barriers, negative clustering energies, molecular dynamics simulation, and kMC results all indicate strong stability of the 2D ferroelectric SACs.

Good stabilities of the ferroelectric substrate and the catalysts themselves are key prerequisites to achieve the goal of controllable catalysis. The stability of ferroelectric α-In_2_Se_3_ monolayer has been unambiguously demonstrated by our simulations (Supplementary Fig. 1) and also by the recent theoretical and experimental studies^35,36,39,40^, but stabilities under harsh evironments for CO_×_RR still need to be addressed. To this end, based on the dissolution potential^57–59^, we have evaluated and confirmed the electrochemical stability of our proposed system under acidic conditions (see Supplementary Table 2).

Different from traditional SACs like Pd@C_3_N_4_, the switchable polarization in ferroelectric catalysts proposed herein provides an extra degree of freedom to modulate and control catalytic reactivities. The average TM-Se bond length, binding energy, and electron transfer are highly polarization dependent (Table 1). Compared with the corresponding catalysts with upwards polarization, TM@P ↓ -In_2_Se_3_ generally have shorter average TM-Se bond length, larger absolute value of binding energy, and more electron transfer, due to the large electrostatic potential difference on In_2_Se_3_ as in metal porphyrazine molecules^60^. The balance between empty and occupied *d* orbitals, caused by the polarization dependent electron transfer, significantly affect the catalytic activity of TM@In_2_Se_3_. Taking Pd@In_2_Se_3_ as an example, the charge is redistributed between the Pd and Se atoms (see Fig. 1f), and the *d* orbitals of Pd atom is depleted. Compared with Pd@P ↑ -In_2_Se_3_, more electron transfer occurs in Pd@P ↓ -In_2_Se_3_, shifting the Pd-*d* orbitals to higher energy closer to the Fermi level. As illustrated in Fig. 1c, the *d* band center of Pd@P ↓ -In_2_Se_3_(−1.45 eV) is higher than that of Pd@P ↑ -In_2_Se_3_ (−4.13 eV), indicating that Pd@P ↓ -In_2_Se_3_ possesses better catalytic ability^46^. Similar phenomena occur in the other five TM@In_2_Se_3_ catalysts (see Fig. 1c) under the same mechanism: the *d* band center of the TM atom is closer to the Fermi level when it is placed on P ↓ -In_2_Se_3_. Note that the position differences of the *d* band center under opposite polarizations for TM@In_2_Se_3_ (TM = Nb, Re) are rather pronounced, while such differences for TM@In_2_Se_3_ (TM = Ni, Pd and Rh) are only moderate. These contrasting results imply that both reaction paths and barriers are affected by the polarization (see a detailed catalytic classification below). Considering that the localized electronic states near the Fermi level lead to high N_2_ catalytic activity in the catalyst Co_2_@GDY (cobalt dimer-anchored graphdiyne)^49^, we expect that the modulation of the electronic structure of TM@In_2_Se_3_ caused by the polarization switching has a major influence on the subsequent CO_2_ hydrogenation process.

Since the catalytic activity of the TM@In_2_Se_3_ systems is polarization dependent, the effective ferroelectric switching of TM@In_2_Se_3_ is essential to achieve controllable catalytic reduction. We reverse the polarization orientation of Pd@In_2_Se_3_ (as a typical example, see Supplementary Figs. 15 and 16) via a three-step concerted mechanism on the most effective kinetic path^38^. We find that the energy barrier of the polarization switching after Pd decoration is 0.29 eV/UC (0.15 eV/UC) for P ↑ to P ↓ (P ↓ to P ↑ ), which is slightly larger than the value for the bare ferroelectric layer (0.13 eV/UC), but it is still accessible to experimental manipulation^38^. It is noted that the ferroelecitrc switching behaviors are not significantly affected by the metal adsorption. Although the local area of metal anoching site is metallized due to the hybridization between the metal and surrounding Se atoms, the semiconduting feature of the whole substrate and the associated ferroelectricity can be well preserved (see Supplementary Fig. 17). Considering that the out-of-plane ferroelectric switching of thin In_2_Se_3_ flakes has been experimentally achieved^40^, it should be feasible to achive similar polarization orientation reversal in TM@In_2_Se_3_ systems.

### Tunable activation of CO_2_ by polarization switching

**Fig. 2:**
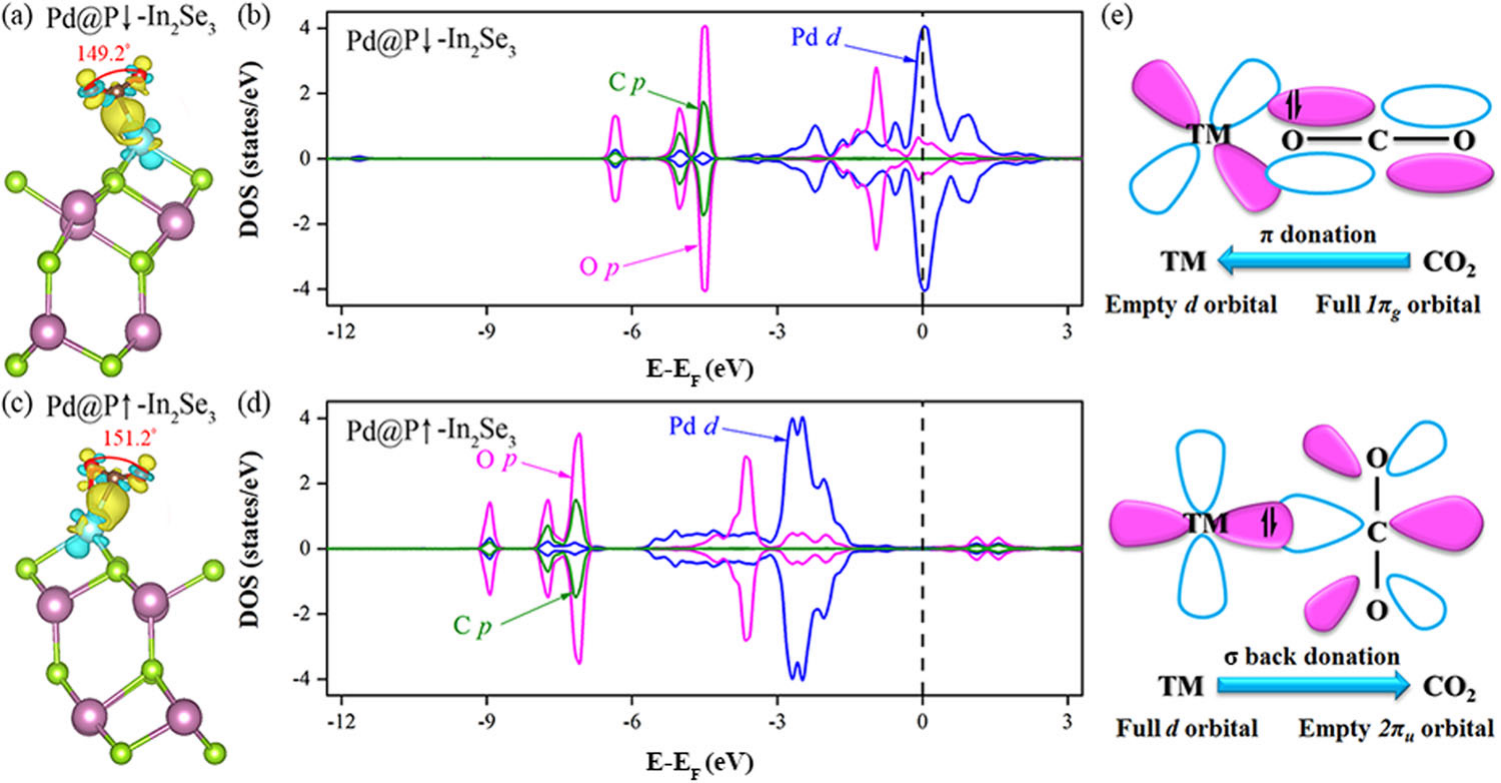
Tunable activation of CO_2_ by polarization switching. The differential charge density plots and partial density of states of CO_2_ adsorbed on Pd@In_2_Se_3_ (**a**, **b** for Pd@P ↓ -In_2_Se_3_, and **c**, **d** for Pd@P ↑ -In_2_Se_3_). **e** Simplified schematic diagrams of CO_2_ bonding to transition metals.

Effective activation of CO_2_ molecule is the key for the subsequent reduction. Based on the structural analysis, almost all the TM@In_2_Se_3_ catalysts could effectively activate CO_2_ molecule (see Fig. 2 and Supplementary Fig. 18) by forming the bidentate C-TM-O species. The only exception is Zr@P ↓ -In_2_Se_3_, where the linear O=C=O structure is well preserved (only bent by 0.8°) because of the monodentate TM-O configuration. Moreover, the binding energies of the catalysts with the activated CO_2_ molecule (−3.39 to −0.76 eV; see Table 1) are comparable to the reported catalysts^55,61,62^. The strong hybridization of O *p* and TM *d* orbitals and the significant charge transfer between TM@In_2_Se_3_ and CO_2_ ensure the inert molecule chemically captured (see Fig. 2b, d, and Supplementary Fig. 19). Note that the degree of CO_2_ activation on two opposite surfaces are obviously different as seen from the angles of ∠OCO (see Table 1), which are generally smaller when the molecule is adsorbed on the surface with polarization downwards. Meanwhile, the activation degree of CO_2_ on TM@In_2_Se_3_ (TM = Nb, Re) is significantly higher than that on TM@In_2_Se_3_ (TM = Ni, Pd, Rh), as indicated by the larger binding energies (−3.39~−2.02 eV vs −1.39~−0.76 eV), more electron transfer (0.61~0.92e vs 0.26~−0.45e), and smaller angles of ∠OCO (131.1°~136.8° vs 146.4°~151.2°).

The distinct behaviours on different ferroelectric surfaces are attributed to the mechanism of CO_2_ catalytic activation, namely coexistence of charge depletion and accumulation between the TM atoms and CO_2_ molecules. The empty TM *d* orbital could accept electrons from the highest occupied molecular orbitals (*1π*_*g*_ orbital) of the CO_2_ molecule, while the occupied TM *d* orbital could back-donate electrons to the lowest unoccupied molecular orbitals (*2π*_*u*_ orbital) of the CO_2_ molecule (Fig. 2e). As indicated in Fig. 2b and d, the *d* orbital occupations of TM atoms on the ferroelectric In_2_Se_3_ layer can be effectively modulated by the switchable polarization. The synergy of electron acceptance, back-donation, and polarization dependent *d* orbital occupation ensures that CO_2_ can be efficiently activated, while the activation degree is ferroelectric controllable in TM@In_2_Se_3_ catalysts^61^. For example, while isolated Pd atom has fully occupied *d* orbital (4*d*^10^), upon adsorption on the In_2_Se_3_ substrate, the Pd atom loses partial *d* electrons to form the Pd-Se bond, and the resulting empty Pd *d* orbitals provide channels for electron acceptance and back donations to activate CO_2_ molecules. Consequently, compared with other TM@In_2_Se_3_ catalysts, where the TM atoms have intrinsic partially occupied *d* orbitals, Pd@In_2_Se_3_ catalyst has a lower degree of CO_2_ activation as indicated by the relatively larger ∠OCO and smaller charge transfer (see Table 1). Furthermore, for TM@In_2_Se_3_ under polarization switching, the density of states (DOS) variations are more evident than the CO_2_ binding energy and electron transfer. This is due to the fact that the DOS variation is directly related with the orbital shift under the polarization flip, such as the *d* band center, as seen in Fig. 1c, while the charge transfer and the CO_2_ binding energy are mainly related to the itinerant electrons induced by the polarization.

### Selectivity for CO_2_RR vs HER

As an important competing side reaction, hydrogen evolution reaction (HER) may significantly restrain the Faradaic efficiency of CO_2_RR by consuming proton–electron pairs from the electrolyte solution^63,64^. To check if CO_2_RR is more favourable, we first calculated Gibbs free energy changes (ΔG) at the first hydrogenation step of CO_2_RR (* +CO_2_ + H ^+^ +e^−^ → OCOH* or OCHO*) and HER (* +H ^+^ +e^−^ → H*). According to the Brønsted–Evans–Polanyi relation^65,66^, reactions with lower ΔG have smaller reaction barriers and consequently are kinetically more favoured.

**Fig. 3:**
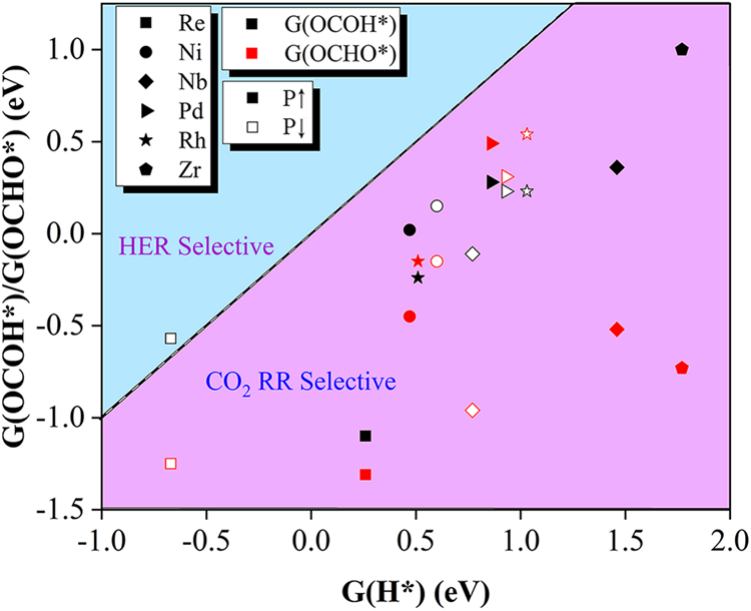
Selectivity for CO_2_RR vs HER. Gibbs free energy changes (ΔG) of initial protonation of CO_2_RR *vs*. HER on TM@In_2_Se_3_ (TM = Ni, Pd, Rh, Zr, Nb and Re). Data points in the purple region denote higher selectivity toward CO_2_RR, while those in the blue region toward HER. Black and red symbols stand for ΔG(OCOH*) vs ΔG(H*) and ΔG(OCHO*) vs ΔG(H*), respectively.

As shown in Supplementary Fig. 20, for the first hydrogenation step of CO_2_RR, Rh@In_2_Se_3_, Pd@In_2_Se_3_, and Re@P ↑ -In_2_Se_3_ prefer to form carboxyl (OCOH*), while Ni@In_2_Se_3_, Nb@In_2_Se_3_, Zr@P ↑ -In_2_Se_3_, and Re@P ↓ -In_2_Se_3_ tend to produce formate (*OCHO). Since CO_2_ molecule cannot be activated on Zr@P ↓ -In_2_Se_3_, it is not considered here. The Gibbs free energy changes (Fig. 3) indicate that all the screened TM@In_2_Se_3_ catalysts have a higher selectivity toward CO_2_RR. These results demonstrate the feasibility of using TM@In_2_Se_3_ catalysts as cathodes for CO_2_RR with a high Faradaic efficiency.

### Tunable CO_2_RR by polarization switching

As a result of polarization dependent *d* band center (see Fig. 1c), CO_2_ molecules are activated to different degrees depending on the ferroelectric surfaces. It is thus expected that the reaction barrier, reaction path, and even the intermediate/final product of CO_2_ reduction will be polarization dependent and controllable via ferroelectric switching.

Considering the complexity of CO_2_ reaction, we comprehensively searched the minimum energy reaction paths of CO_2_RR on each TM@In_2_Se_3_ (TM = Ni, Pd, Rh, Zr, Nb and Re) catalyst (Supplementary Fig. 20). Table 2 summarizes the effects of the polarization switching on the CO_2_RR, from the aspects of potential determining steps, limiting potential (*U*_*l*_), and final product.

**Table 2:** Potential determining steps (PDS), limiting potentials (*U*_*l*_, V vs RHE), and final product of CO_2_RR with TM@In_2_Se_3_ catalysts.

**Catalyst**	**PDS**	***U***_***l***_	**Product**
Ni@P ↓ -In_2_Se_3_	HOCHO*→CHO*	−0.36	CH_4_
Ni@P ↑ -In_2_Se_3_	HOCHO*→CHO*	−0.47	CH_4_
Pd@P ↓ -In_2_Se_3_	CO*→CHO*	−0.77	CH_4_
Pd@P ↑ -In_2_Se_3_	CO*→CHO*	−0.87	CH_4_
Rh@P ↓ -In_2_Se_3_	CO*→CHO*	−0.43	CH_4_
Rh@P ↑ -In_2_Se_3_	CHO*→CH_2_O*	−0.26	CH_4_
Zr@P ↓ -In_2_Se_3_	HOCHO*→CHO*	−2.33	CH_4_
Re@P ↓ -In_2_Se_3_	OH*→H_2_O*	−0.65	CH_3_OH
Re@P ↑ -In_2_Se_3_	COH*→C*	−0.85	CH_4_
Nb@P ↓ -In_2_Se_3_	OH*→H_2_O*	−0.64	CH_3_OH
Nb@P ↑ -In_2_Se_3_	OCHO*→HOCHO*	−0.58	HOCHO

Depending on the effects of the reversible polarization, the ferroelectric catalysts for CO_2_RR can be classified into three categories: (i) TM@In_2_Se_3_ (TM = Ni, Pd, Rh), for which the polarization switching changes the reaction barriers, but not the reaction paths and the intermediate/final products; (ii) Zr@In_2_Se_3_, for which the ferroelectric switching can reactivate the stuck CO_2_ reduction; (iii) TM@In_2_Se_3_ (TM = Nb, Re), for which all the reaction paths, reaction barriers, and final products are polarization dependent. This classification is well consistent with *d* band center analysis (Fig. 1c), as well as the binding energy, charge transfer, and activation degree of CO_2_ as listed in Table 1, which indicates clear underlying relationship among these material properties and catalytic performance.

### Tunable limiting potential of CO_2_RR (on TM@In_2_Se_3_, TM = Ni, Pd, Rh)

**Fig. 4:**
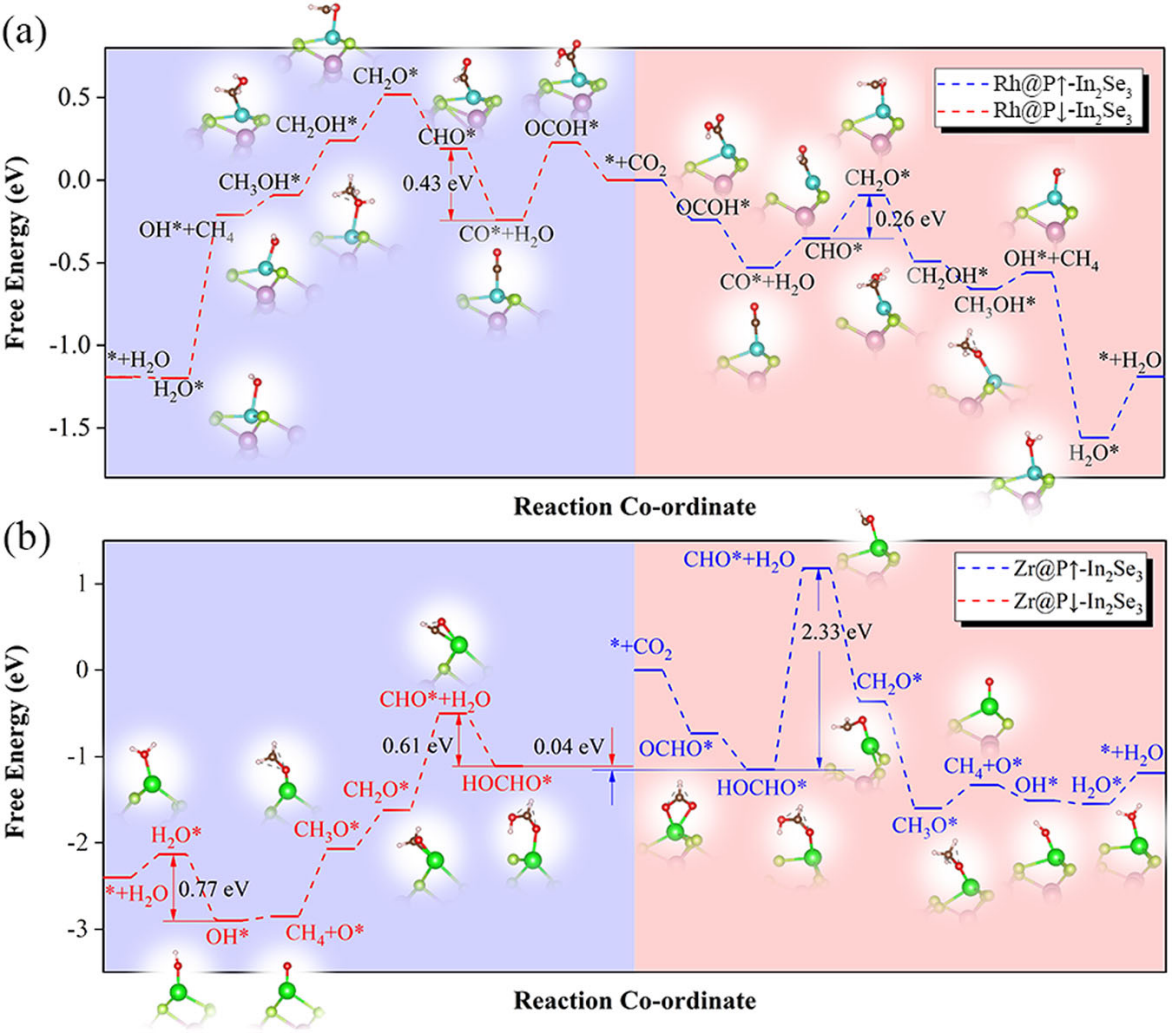
CO_2_RR paths on Rh@In_2_Se_3_ and Zr@In_2_Se_3_. The free-energy profile for the CO_2_ electrochemical reduction reactions along the minimum energy path at 0 V (vs. RHE) on **a** Rh@In_2_Se_3_ and **b** Zr@In_2_Se_3_. The insets show the optimized configurations of the intermediates. The pink (light blue) shaded area indicates the catalytic reaction when the polarization is pointing upwards (downwards). This color scheme is also used in Fig. 5.

Since the activation degree of CO_2_ on the ferroelectric surface depends on polarization direction due to the adjusted empty and occupied *d-*orbitals (shifted *d* band center), the reaction barrier of the CO_2_ hydrogenation process is also affected. Taking the Rh@In_2_Se_3_ catalyst as an example (see Fig. 4a), though the configurations of activated CO_2_ (with C atom connecting with Rh atom) under different polarization directions are the same, the energy barriers are obviously different: the reaction of CO_2_* →OCOH* is downhill on Rh@P ↑ -In_2_Se_3_, but uphill on Rh@P ↓ -In_2_Se_3_. In the subsequent hydrogenation steps, the polarization reversal does not change the most favourable path of CO_2_RR, both following the paths of CO_2_* →OCOH* →CO* →CHO* →CH_2_O* →CH_2_OH* →CH_3_OH* →OH* →H_2_O*, with the final product of CH_4_. However, the reaction barrier for each step, the potential-determining step (PDS), and the limiting potential are all different and polarization dependent. On Rh@P ↓ -In_2_Se_3_, the PDS of CO_2_ reduction is CO* →CHO* with a limiting potential of −0.43 V *vs*. RHE; in contrast, the corresponding PDS on Rh@P ↑ -In_2_Se_3_ is the protonation of CHO* to CH_2_O* with the estimated *U*_*l*_ of −0.26 V *vs*. RHE. Obviously, the reaction barrier of CO_2_ reduction is polarization tunable via ferroelectric switching. Notably, the limiting potential (−0.26 eV *vs*. RHE) is better than most reported CO_2_RR catalysts (listed in Supplementary Table 6). Therefore, Rh@In_2_Se_3_ is potentially an efficient electrochemical catalyst for CO_2_RR with controllable performance. Similar phenomena of polarization dependent catalysis also exist in Ni@In_2_Se_3_ and Pd@In_2_Se_3_ (Table 2 and Supplementary Fig. 21). The relatively weaker but still obvious ferroelectric dependent CO_2_ reduction relates with the moderately polarization dependent *d* band center (−1.98~−1.16 eV at P ↓ ; −4.13~−3.16 eV at P ↑ , Fig. 1c) and modest activation degree of CO_2_, as indicated by the moderate CO_2_ binding energies (*E*_b-CO2_, 0.79-1.39 eV) and mild charge gained by the adsorbed CO_2_ molecule (*Q*_CO2_, 0.26-0.45e) (Table 1).

### Reactivating stuck catalytic reaction of CO_2_RR (on Zr@In_2_Se_3_)

Zr@P ↓ -In_2_Se_3_ cannot activate CO_2_ molecules, it is thus unsuitable for CO_2_ reduction. In contrast, CO_2_ can be activated on Zr@P ↑ -In_2_Se_3_ with two oxygen atoms bridging over Zr atom (Supplementary Fig. 18i, j). With more protons, it follows the hydrogenation steps of CO_2_RR as * + CO_2_ + H ^+^ +e^−^ → OCHO* and OCHO* +H ^+^ +e^−^ → HOCHO*; both of these steps are downhill, indicating that the reduction can react easily. However, further hydrogenation step is prevented by the strong binding strength of the intermediate HOCHO* (∆G = 2.33 eV, Fig. 4b) due to significant electron transfer and over-activations (1.13 e between CO_2_ and catalyst, 126.2° of **∠**OCO as shown in Table 1). Based on the Sabatier principles, such strong binding energies make further reduction only possible at very negative potentials. Further hygenation is thus prehibitted.

Since polarization can modulate interaction strength as indicated above, it is expected that the binding strength of the intermediate HOCHO* can be weakened when the polarization direction of In_2_Se_3_ is reversed, so that the catalytic reduction can proceed. Indeed, we found that when polarization direction is reversed from up to down at this step, the barrier decreases (∆G = 0.61 eV for the reduction of HOCHO* to CHO* on Zr@P ↓ -In_2_Se_3_). More importantly, HOCHO* on the Zr@P ↓ -In_2_Se_3_ is merely 40 meV higher than that on Zr@P ↑ -In_2_Se_3_, which indicates that the polarization switching is relatively easy to achieve. The conclusion holds true under dilute Zr concentrations, while the local ferroelectric switching around HOCHO* is prohibited due to the higher energy barrier (see Supplementary Fig. 22). Additionally, according to the subsequent hydrogenation steps, the *U*_*l*_ for Zr@P ↓ -In_2_Se_3_ is −0.77 V *vs*. RHE, which is significantly lower than that for Zr@P ↑ -In_2_Se_3_ (−2.33 V *vs*. RHE). Moreover, the H_2_O* at Zr@P ↓ -In_2_Se_3_ could be removed spontaneously, in contrast to the 0.36 eV energy barrier for the dehydration process on Zr@P ↑ -In_2_Se_3_.

Therefore, though CO_2_ molecule cannot be activated on Zr@P ↓ -In_2_Se_3_ for reduction, the polarization reversal can reactivate the catalytic reduction on Zr@P ↓ -In_2_Se_3_ with a reasonable limiting potential. During the hydrogenation process, appropriate polarization switching could either reactivate the CO_2_ reduction or accelerate the reaction rate by reducing the reaction barriers.

### Tuning CO_2_RR path and final product (on Nb@In_2_Se_3_ and Re@In_2_Se_3_)

In contrast to the aforementioned TM@In_2_Se_3_ (TM = Ni, Pd, Rh), the activations of CO_2_ molecule are much deeper on Nb@In_2_Se_3_ and Re@In_2_Se_3_, as evidenced by the smaller bond angle ∠OCO (Table 1), much larger *E*_b-CO2_ values (2.02-2.54 eV), and more pronounced charge transfer between CO_2_ and the catalyst (*Q*_CO2_, 0.61-0.92e). The huge differences of *d* band center (−2.52~−2.52 eV at P ↓ ; −7.03~−6.72 eV at P ↑ , see Fig. 1c) render the catalytic reaction more sensitive to the polarization switching, leading to different reaction paths and even different final products according to the Sebastian principles.

**Fig. 5:**
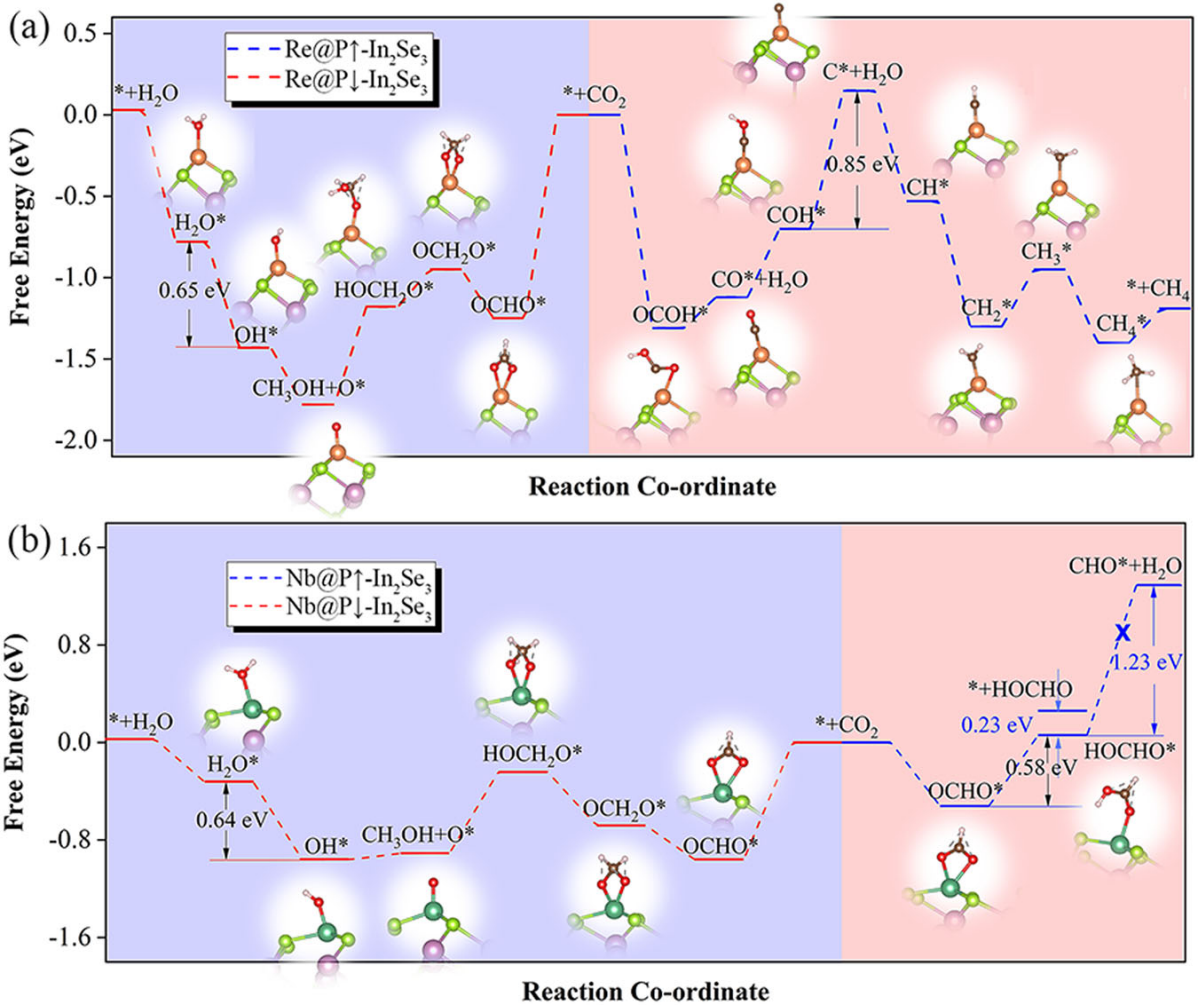
CO_2_RR paths on Re@In_2_Se_3_ and Nb@In_2_Se_3_. Free energy profile for CO_2_ electrochemical reduction reactions along the minimum energy path at 0 V (*vs*. RHE) on **a** Re@In_2_Se_3_ and **b** Nb@In_2_Se_3_. The insets show the optimized configurations of the intermediates.

Indeed, the polarization switching in the catalysts of Nb@In_2_Se_3_ and Re@In_2_Se_3_ could partially or even totally alter the CO_2_RR path, thus leading to the different final products. As illustrated in Fig. 5a, for CO_2_RR on Re@P ↓ -In_2_Se_3_ catalyst, the minimum energy path is CO_2_* →OCHO* →OCH_2_O* →HOCH_2_O* →O* →OH* →H_2_O*, the final product is methanol, and the PDS is OH* →H_2_O* with U_*l*_ of 0.65 eV; In sharp contrast, the reaction paths on Re@P ↑ -In_2_Se_3_ is CO_2_* →OCOH* →CO* →COH* →C* →CH* →CH_2_* →CH_3_* →CH_4_*, the final product is methane, and the PDS is COH* →C* with U_*l*_ of 0.85 eV. Similar CO_2_RR phenomena were also observed on Nb@In_2_Se_3_ catalyst. The most favourable path on Nb@P ↓ -In_2_Se_3_ surface is CO_2_* →OCHO* →OCH_2_O* →HOCH_2_O* →O* →OH* →H_2_O*, producing methanol with U_*l*_ of 0.64 eV; in comparison, the CO_2_* →OCHO* →HOCHO* path on Nb@P ↑ -In_2_Se_3_ produces methanoic acid with U_*l*_ of 0.58 eV (Fig. 5b).

**Fig. 6:**
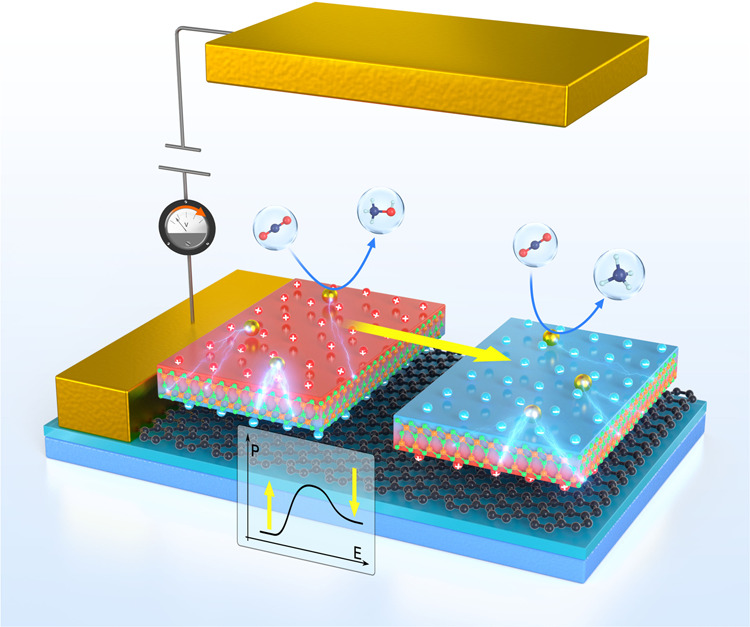
Schematic diagram of ferroelectric controllable CO_2_RR. Here, metal anchored α-In_2_Se_3_ monolayer is placed between the electrodes to achieve ferroelectric switching and controllable catalysis, tuned by the reversal of the bias direction.

Note that selective generation of desired chemical fuel products has been pursued in the development of electrochemical CO_2_RR catalyst over decades^5^, our strategies via feasible ferroelectric switching can achieve the goal without complex and high-cost steps that use different catalysts or electrolytes to produce desired fuels^67–70^. As shown in Fig. 6, our proposed ferroelectric SACs base on experimentally available In_2_Se_3_ and unique polarization dependent catalytic performance thus open an avenue for controllable CO_2_ reduction.

## Discussion

In conclusion, by comprehensive DFT computations, we have screened out six TM@In_2_Se_3_ (TM = Ni, Nb, Pd, Re, Rh and Zr) as effective ferroelectric catalysts for electrochemical CO_2_RR. We found that the polarization reversal of ferroelectric In_2_Se_3_ monolayer can adjust the empty/occupied *d* orbitals of adsorbed TM atom and the energy of the *d* electrons, thereby tuning the catalytic performance for CO_2_RR, including the degree of CO_2_ activation, limiting potential, reaction path, and final product, even reactivating stuck catalytic reduction. Especially, the Rh@P ↑ -In_2_Se_3_ catalyst is indentified as a highly efficient electrochemical catalyst for CO_2_RR due to the fairly low limiting potential (<0.5 V)^71^. Moreover, the Re@In_2_Se_3_ and Nb@In_2_Se_3_ catalysts can realize selective generation of different desired products with the same catalyst via polarization switching, which opens a path toward ferroelectric controllable catalysis. These SACs based on 2D ferroelectric materials hold great promise for improving catalytic activity and selectivity for electrochemical CO_2_RR.

## Methods

### Computational details

Our density functional theory (DFT) computations are performed with the Vienna ab initio simulation package (VASP) code^72,73^. The spin-polarized generalized gradient approximation (GGA) in the form of Perdew − Burke − Ernzerhof (PBE) treats the exchange − correlation interactions, while the frozen-core projector augmented wave (PAW) approximation describes the interaction between the ion and electron^74–76^. The van der Waals (vdW) interactions are described with the DFT-D3 method in Grimme’s scheme^77^. The models with one TM atom anchored 2 × 2 In_2_Se_3_ are built as the potential catalysts for the simulations, since it corresponds to the coverage of 25% with the optimal adsorption energies, as shown in Supplementary Fig. 23. Four TM atoms uniformly distributed on the hexagonal centers of 4 × 4 In_2_Se_3_ supercell act as the catalytic active sites. More than 20 Å vacuum space perpendicular to the surface is added to avoid interactions between periodic slabs. The dipole correction is taken into account for all the asymmetric structures^78^. The 2D Brillouin sampling is presented with a 4 × 4 × 1 gamma-centered Monkhorst-pack *k*-mesh. The cutoff energy for plane-wave basis sets is 500 eV. The convergence thresholds for force and total energy are 10^−2^ eV/Å and 10^−5^ eV, respectively. Moreover, the Gibbs free energy calculation is operated with computational hydrogen electrode (CHE) model^79^, and the solvent effect is considered with the implicit solvent model implemented in VASPsol^27,61^. The site-specific charge differences are obtained using Bader analysis. Besides the standard PBE calculations and the models of TM@2 × 2 In_2_Se_3_, we also reexamined the chemical reactions by using larger supercells (TM@4 × 4 or 6 × 6 In_2_Se_3_, Supplementary Fig. 24) or different exchange functions (PBE + U or RPBE; Supplementary Figs. 25 and 26). All these approaches lead to the same conclusion about the ferroelectric controlled CO_2_ reduction, with the key numerical results only slightly different, indicating the robustness of our conclusion. More details of the simulations can be found in the Supplementary Information.

## Supplementary information

supporting information

Peer Review File

## Data Availability

Source data are provided with this paper. The data used in this study are presented in the text and Supplementary Information. Additional data and information are available from the corresponding author upon reasonable request. Source data are provided with this paper.

## Source data

source data
