# Supplementary material for: Controllable CO2 electrocatalytic reduction via ferroelectric switching on single atom anchored In2Se3 monolayer
Source: Nat Commun. 2021 Aug 26;12:5128. doi: 10.1038/s41467-021-25426-5 (PMC8390745; doi:10.1038/s41467-021-25426-5)
Supplement: Supplementary file 1 — supporting information [file 41467_2021_25426_MOESM1_ESM.pdf]

Controllable CO_2 Electrocatalytic Reduction via Ferroelectric Switching on Single Atom Anchored In_2Se_3 Monolayer

Lin Ju[1, 2, ★], Xin Tan[3, ★], Xin Mao[4], Yuantong Gu,[1, 5, 6] Sean Smith[3], Aijun Du[4, 5], Zhongfang Chen[7], Changfeng Chen[8] and Liangzhi Kou[1, 5*]

[1]School of Mechanical, Medical and Process Engineering Faculty, Queensland University of Technology, QLD 4001, Australia

[2]School of Physics and Electric Engineering, Anyang Normal University, Anyang, 455000, China

[3]Integrated Materials Design Laboratory Research School of Physics, The Australian National University Canberra ACT 2601, Australia

[4]School of Chemistry and Physics, Queensland University of Technology, QLD 4001, Australia

[5]Center for Materials Science, Queensland University of Technology, Brisbane, QLD, 4001, Australia

[6]Centre for Biomedical Technologies, Queensland University of Technology, Brisbane, QLD 4000, Australia

[7] Department of Chemistry, University of Puerto Rico, Rio Piedras Campus, San Juan, Puerto Rico, 00931, United States

[8]Department of Physics and Astronomy, University of Nevada, Las Vegas, Las Vegas, Nevada, 89154, United States

The intrinsic stability of α-In_2Se_3 and structural polymorphs

The In-Se compounds have many possible structural polymorphs, such as the phases of α, β, γ, δ, κ-In_2Se_3, which have been determined by X-ray diffraction and TEM.[1] However, α-In_2Se_3 is the ground-state phase and is stable at the room temperature from both theoretical and experimental perspectives, as elaborated below.

Theory: To examine the stabilities of different structural polymorphs, we calculated the total energies of six possible phases of the In_2Se_3 monolayer (**Figure S1**), including the β', β, α and α', zincblende, and wurtzite phases. The α-In_2Se_3 monolayers with ferroelectric polarizations have the lowest total energies, consistent with the recent theoretical work by Ding *et al.*[2], indicating α-In_2Se_3 to be the most stable phase.

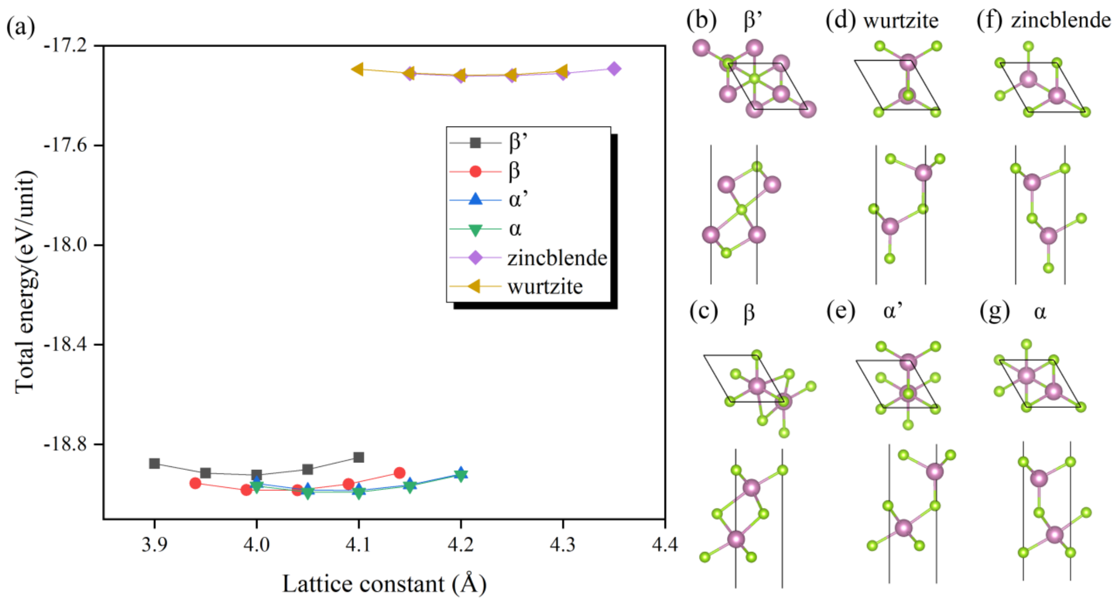

Figure S1 (a) Calculated total energy versus lattice constant for six In_2Se_3 monolayer phases. (b)-(g) Top and side views of these six In_2Se_3 monolayers, among which the structures shown in (b), (d), and (f) are derived from the fcc, wurtzite, and zincblende crystals, respectively.

Experiments: Different phases In_2Se_3 (α, β, γ, δ, κ-phase) have been experimentally synthesized,[1] but under distinct fabrication conditions. Past reports have explicitly indicated that α-In_2Se_3 is the room-temperature phase,[3, 4] while β, γ, δ -phases are high-temperature phases.[5] Phase transformation can be achieved *via* the path of $\alpha \xrightarrow{200^\circ C} \beta \xrightarrow{520^\circ C} \gamma \xrightarrow{730^\circ C} \delta$,[1] which also indicates that α phase is the stable room-temperature structure. This point has been further verified by the recent synthesis of α-In_2Se_3 layers that have been taken to fabricate ferroelectric devices.[6, 7, 8] Cui *et al.* have pointed out that the cooling rate is critical

for obtaining the α phase, which is stable at room temperature.[9] Therefore, α-In_2Se_3 is rather stable at room temperature and can be well prepared by controlling the synthesizing temperature.

Total energy of possible In/Se phases in the presence of metal adatoms and in the electrochemical environment

In order to check if α-In_2Se_3 is still the global minimum energy phase in the presence of metal adatoms and in the electrochemical environment, we calculated the total energies of other four phases (β', β, wurtzite, and zincblende) with presences of Pd, H* or OH*. As shown in **Figure S2**, for all the studied cases, the α-In_2Se_3 has the lowest total energy among these five phases.

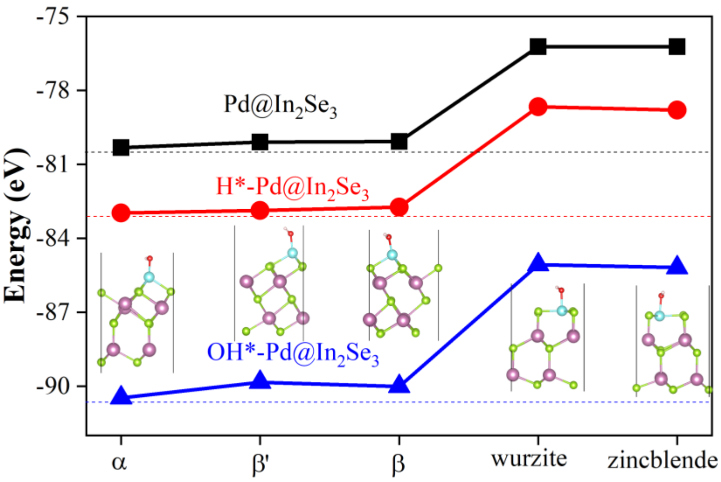

Figure S2 Calculated total energy for: (a) α, β', β, wurtzite, and zincblende phases of Pd@In_2Se_3; (b) H* and (c) OH* adsorbed Pd@In_2Se_3. The structural configurations of OH*-Pd@In_2Se_3 are inserted as the insets while these of H*-Pd@In_2Se_3 and Pd@In_2Se_3 are not shown since they are similar to OH* adsorbed counterparts.

For other phases with different In/Se ratio, we only considered the InSe monolayer since it was experimentally synthesized. [10, 11] Due to the different species and number of atoms, we cannot simply use the total energies to make a comparison between Pd@InSe and Pd@α-In_2Se_3. Herein the cohesive energy is employed instead.

The cohesive energy of Pd@InSe ($E_{f,\mathrm{Pd@InSe}}$) is defined as follows:

$$E_{f,\mathrm{Pd@InSe}} = (E_{\mathrm{Pd@InSe}} - n_1 E_{\mathrm{In-bulk}} - n_2 E_{\mathrm{Se-bulk}} - E_{\mathrm{pd-bulk}})/N \qquad \text{(ES1)}$$

where $E_{In-\mathrm{bulk}}$, $E_{se-\mathrm{bulk}}$ and $E_{\mathrm{pd-bulk}}$ are the energies of the In and Se atoms in their most stable bulk structures, respectively; n_1, and n_2 are the numbers of the In and Se atoms in Pd@InSe; N is the total number of atoms in Pd@InSe. With the same method, the cohesive

energies of Pd@In_2Se_3 ($E_{f,\mathrm{Pd@In_2Se_3}}$), Pd@InSe ($E_{f,\mathrm{H*-Pd@InSe}}$, $E_{f,\mathrm{OH*-Pd@InSe}}$) and Pd@α-$In_2Se_3$ with H* or OH* adsorptions ($E_{f,\mathrm{H*-Pd@In_2Se_3}}$, $E_{f,\mathrm{OH*-Pd@In_2Se_3}}$) could be calculated as well. As listed in **Table S2**, α-In_2Se_3 always has the lower formation energy than InSe.

All these results above indicate that, α-In_2Se_3 is the global minimum energy phase, even in the presence of adatoms and in electrochemical environment.

Table S1 Calculated formation energy (in eV/atom) of pure Pd@InSe and α phase of Pd@In_2Se_3, as well as these with H* or OH* adsorption.

		H*-adsorbed	OH*-absorbed
Pd@InSe	-0.456	-0.358	-0.324
Pd@In_2Se_3	-0.530	-0.452	-0.445

Stability of α-In_2Se_3 under harsh environments

We have evaluated the electrochemical stabilities of α-In_2Se_3 monolayer by the dissolution potential U_{diss}, [12, 13, 14] which is defined as

$$U_{diss} = U^{\circ}_{diss}(bulk) - {E_f}/{ne} \quad \text{(ES2)}$$

where $U^{\circ}_{diss}(bulk)$ and n are the standard dissolution potential of In/Se bulk and the number of electrons involved in the dissolution, respectively, which can be obtained from the NIST database.[15] E_f is the formation energy of In/Se atom in the In_2Se_3 monolayer, given by:

$$E_{f-Se} = (E_{In_2Se_3} - 2E_{In-bulk} - 3E_{Se-bulk})/3 \quad \text{(ES3)}$$

$$E_{f-In} = (E_{In_2Se_3} - 2E_{In-bulk} - 3E_{Se-bulk})/2 \quad \text{(ES4)}$$

where $E_{In-bulk}$, $E_{se-bulk}$ are the respective total energies of the In and Se atoms in their most stable bulk structures, $E_{In_2Se_3}$ is the total energy of the In_2Se_3 monolayer. According to this definition, materials with $U_{diss} > 0$V *vs* SHE are regarded as electrochemically stable under acidic conditions. The U_{diss} of both In and Se in the In_2Se_3 monolayer are positive (see **Table S3**), indicating the electrochemical stability of the In_2Se_3 monolayer.

Table S2 Formation energy (E_f) and dissolution potential (U_{diss}) of metals, total energy of In/Se atoms in their bulk phase (E_x, x=In and Se), number of transferred electrons (n) during the dissolution, and standard dissolution potential ($U^{\circ}_{diss}(bulk)$) of In/Se atoms.

Elements	E_x (eV)	E_f (eV)	n	$U^{\circ}_{diss}(bulk)$ (V)	U_{diss} (V)
In	-3.90	-1.45	3	-0.34	0.14
Se	-2.75	-0.97	2	0.74	1.23

We note that there are two α-In_2Se_3 phases which share very similar atomic structures and are almost energetically degenerate, the further calculations show that they have the same CO_2RR performance as shown in **Figure S3**.

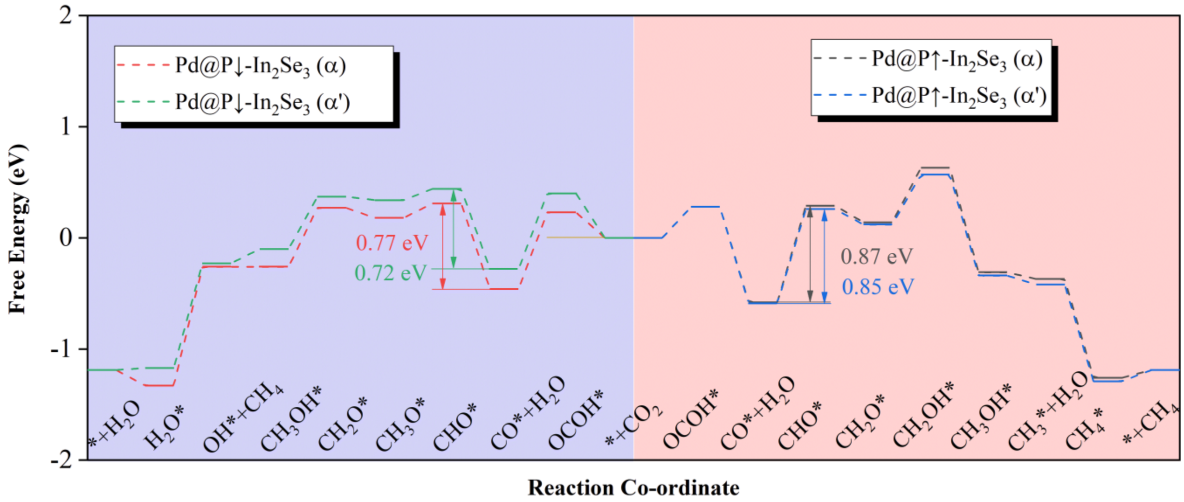

Figure S3 Comparison of CO_2RR pathways on α and α' phases of Pd@In_2Se_3.

Table S3 Parameters for the CO_2 adsorbed In_2Se_3 systems: binding distance of S_{ad}-O and S_{ad}-C ($l_{Sad\text{-}O}$ and $l_{Sad\text{-}C}$, in Å) and bond angle (∠OCO in °).

Configuration	S_{ad}	$l_{Sad\text{-}O}$	$l_{Sad\text{-}C}$	∠OCO
P↓- In_2Se_3	S	3.34	3.12	178.8
P↑- In_2Se_3	I	4.12	4.00	178.6

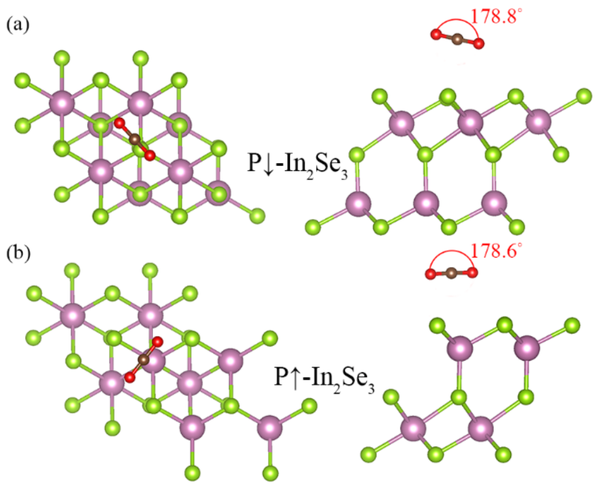

Figure S4 Top and side views of optimized configuration of CO_2 adsorbed on In_2Se_3 monolayer with down (a) and up (b) polarization.

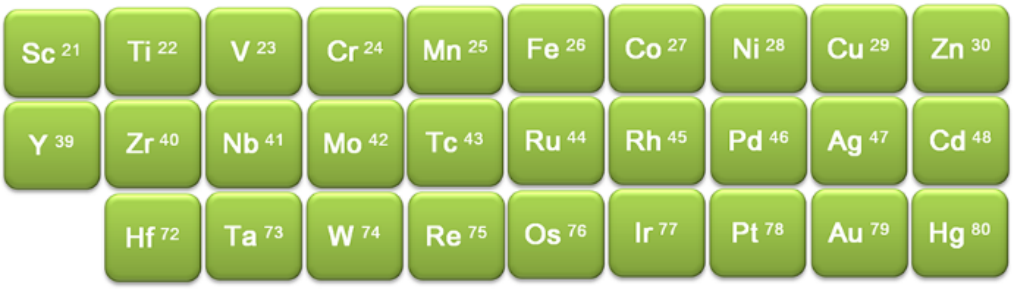

Figure S5 These 29 kinds of transition metals were chosen to modify the In_2Se_3 surface to increase its reactivity.

Metal substituted In_2Se_3

To determine possible metal substation on the In sites, we have simulated the formation of In vacancies. The formation energy of In vacancy and the diffusion barriers of an In atom removal from In_2Se_3 (see Figure S6) have been calculated, since they are the prerequisites for metal atom substitution to occupy the In vacancy sites. Our computational results show that the formation energies of In vacancy are 1.33 and 1.52 eV, respectively, for P↑- and P↓-In_2Se_3. These high formation energies indicate the difficulty to form In vacancy from thermodynamic point of view. Moreover, the energy barriers of an In atom diffusion from the subsurface to the top-surface (to create the vacancy) are 2.68 and 2.94 eV, respectively, for P↑- and P↓-In_2Se_3, which suggest a very small possibility to form In vacancy from the kinetic point of view.

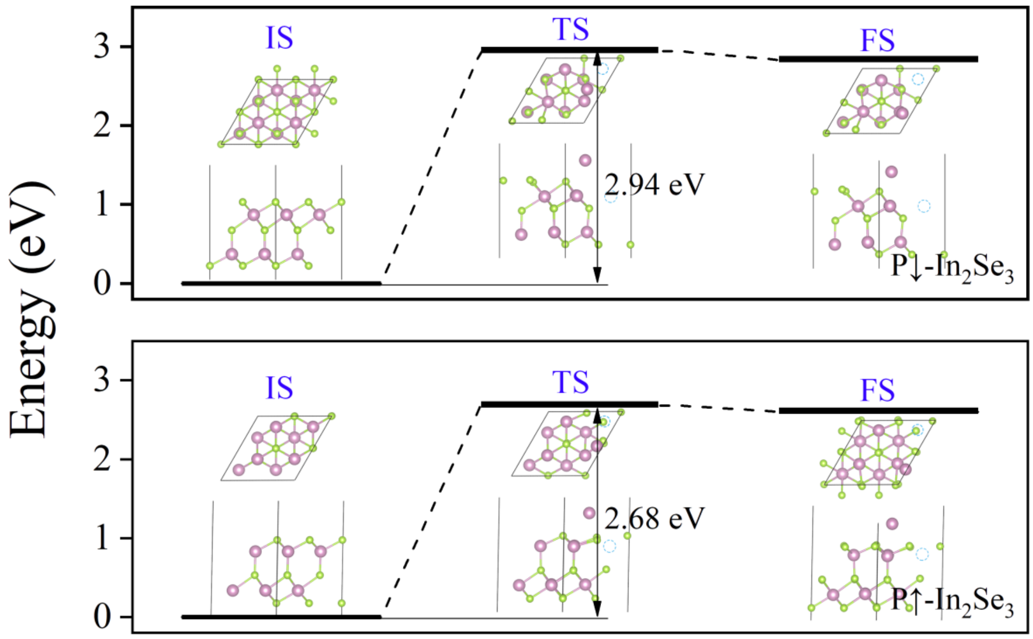

Figure S6 The calculated energy barriers of an In atom diffusion from the subsurface to the top-surface for P↑- and P↓-In_2Se_3. The insets show the optimized structures of initial states (IS), the transition states (TS), and final states (FS) of In atom diffusion. The blue broken circle represents the initial position of the In atom.

In addition, we find that the metal substituted In_2Se_3 is not suitable for the electrocatalytic CO_2RR, even if we do not consider the difficulties of In vacancy formation and metal substitutions. According to the free-energy profile for CO_2 electrochemical reduction reactions along the minimum energy path at 0 V (vs. RHE) on Pd substituted P↑- and P↓-In_2Se_3 (namely Pd substitutes the In vacancy), the energy barriers of $CO_2 + * \rightarrow OCOH^*$ are

up to 1.17 and 1.88 eV on Pd-doped P↑- and P↓-In_2Se_3, respectively, due to the full occupations of Pd-d orbitals (see **Figure S7**), leading to the low CO_2RR activities of these catalysts.

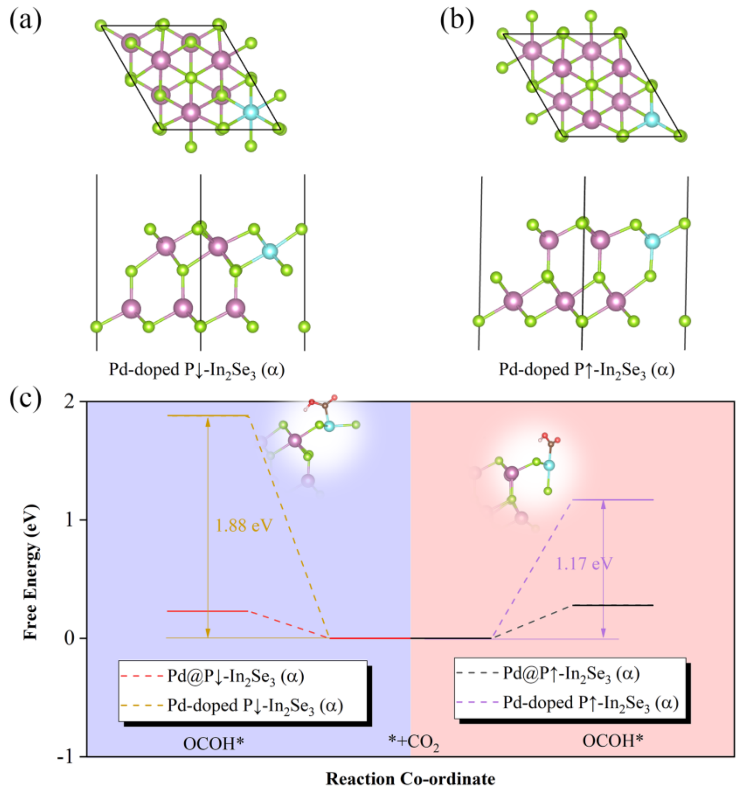

Figure S7 Top and side views of Pd-doped In_2Se_3 monolayer with (a) downward and (b) upward polarization (P↓ and P↑). (c) The free-energy profile for the first hydrogenation step of CO_2RR (COOH*) on Pd@In_2Se_3 and Pd-doped In_2Se_3, respectively.

Overall, we conclude from our calculations and analysis that (i) In vacancies in In_2Se_3 are hard to form due to the high formation energies and diffusion barriers, making metal substituted In_2Se_3 rare in reality and (ii) metal substituted In_2Se_3 is unsuitable for CO_2RR. Therefore, we focus our discussions in this work on metal anchored In_2Se_3 on the surface.

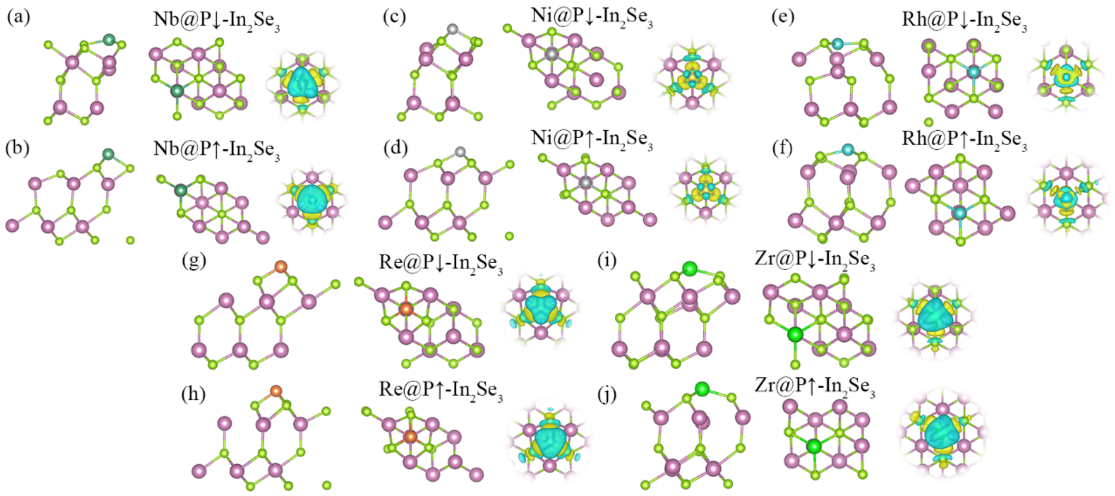

Figure S8 Side and top and views of optimized configuration of TM@P↓-In_2Se_3 (a, c, e, g, and i) and TM@P↑-In_2Se_3 (b, d, f, h, and j). The 3D differential charge density plots of TM@ In_2Se_3 are also shown in this figure, which are obtained with these optimized configurations. The isosurfaces are 0.005 e/Å^3.

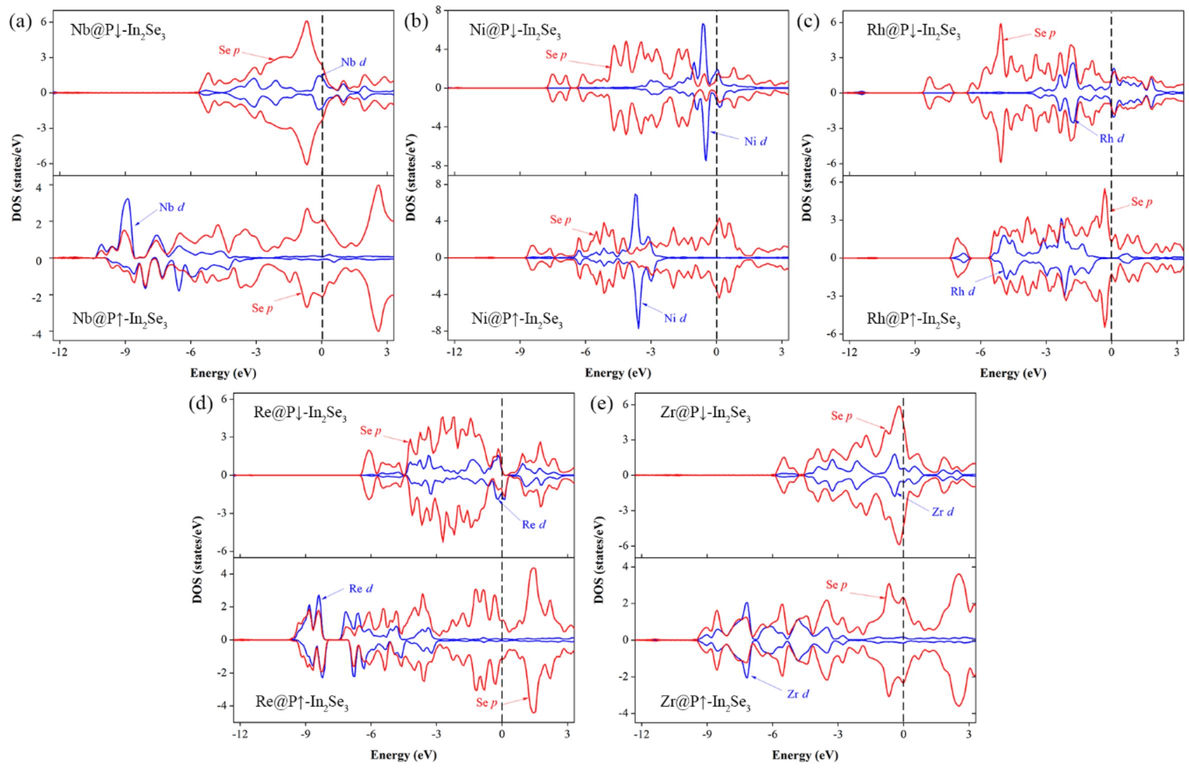

Figure S9 The partial density of states of TM@In_2Se_3 systems including TM@P↓-In_2Se_3 and TM@P↑-In_2Se_3 configurations (TM= (a) Nb, (b) Ni, (c) Rh, (d) Re and (e) Zr)).

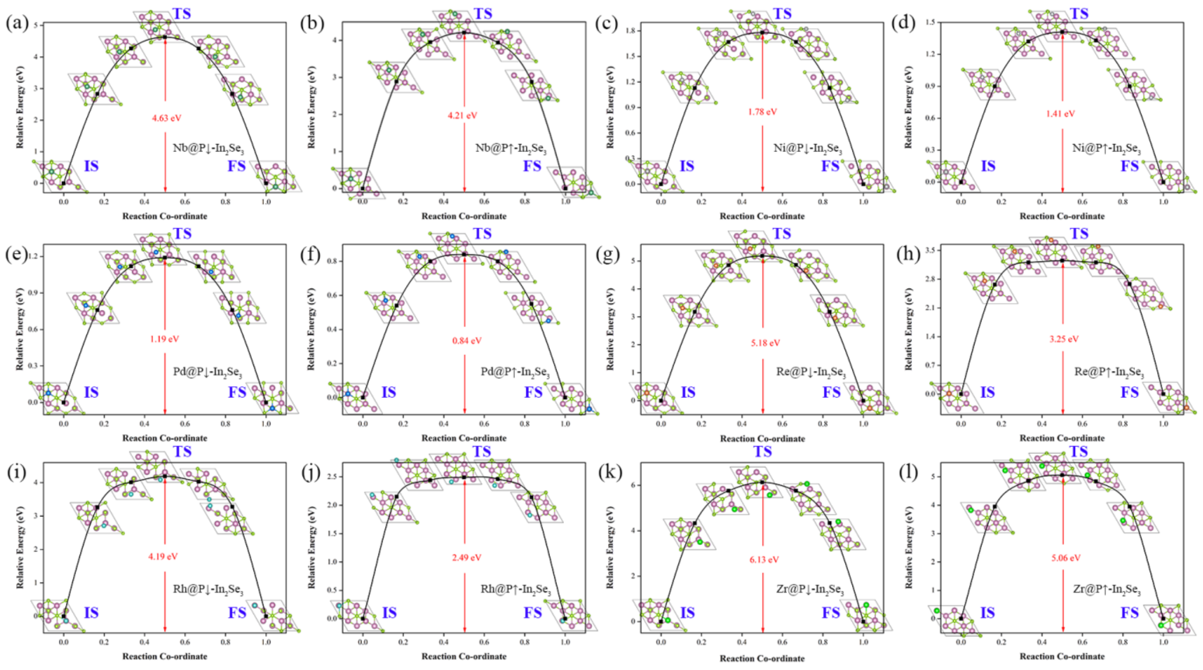

Figure S10 Atomic configurations for the diffusion of the TM (TM=Nb, Ni, Pd, Rh, Re and Zr) single atoms from one favourable adsorption site to its neighbouring one at the TM@P↓-In_2Se_3 and TM@P↑-In_2Se_3 surfaces, including initial state (IS), transition state (TS) and final state (FS). The energy is given with respect to IS. These results are based on the climbing image nudged elastic band (CI-NEB) method.

The $E_{b,\text{precursor}}$ *vs.* $E_{b,\text{SAC}}$

To facilitate the judgment of the binding strength, we take the binding energies of TM atom in the corresponding molecular precursors for the SACs as the references. For Pd based SAC, the energy difference $E_{\Delta b}$ between binding energy of Pd single atom in $Pd(hfac)_2$ (molecular precursor for Pd SAC[16] ($E_{b,\text{Pd(hfac)}_2}$) and the one in $Pd@In_2Se_3$ ($E_{b-\text{Pd}}$), is defined to reflect binding strength of Pd atom in $Pd@In_2Se_3$.

$$E_{\Delta b} = E_{b-\text{Pd}} - E_{b,\text{Pd(hfac)}_2} \tag{ES5}$$

The binding energy of Pd atom in $Pd(hfac)_2$ ($E_{b,\text{Pd(hfac)}_2}$) is defined as below:

$$E_{b,\text{Pd(hfac)}_2} = E_{\text{Pd(hfac)}_2} - 2E_{\text{hfac}*} - E_{\text{Pd}} \tag{ES6}$$

where $E_{\text{Pd(hfac)}_2}$ and E_{Pd} are the total energy of $Pd(hfac)_2$ and isolated Pd atom.

$E_{\text{hfac}*}$ is defined as:

$$E_{\text{hfac}*} = E_{\text{HfacH}} - \frac{1}{2}E_{H_2} \tag{ES7}$$

where E_{HfacH} and E_{H_2} are total energy of the hexafluoroacetylacetonate and hydrogen molecules.

The calculated values of $E_{\Delta b}$ are -0.70 and -0.15 eV for Pd@P↓-In_2Se_3 and Pd@P↑-In_2Se_3, respectively. We can thus conclude that: 1) the adsorption of Pd atom on In_2Se_3 is more stable, than that of $Pd(hfac)_2$; 2), the $Pd@In_2Se_3$ could be synthesized by using $Pd(hfac)_2$ as the molecular precursors.

Using the same methods, we also calculated the energy differences $E_{\Delta b}$ for Rh and Ni based catalysts, see **Table S4**, all of them are negative, indicating the stability and possible synthesis based on the molecular precursors.

For Re, Zr and Nb, the corresponding molecular precursors for single atom catalysts synthesis are not found from the previous literatures, but we noticed that the metal rods were used as the sources for the fabrications of single atom catalysts,[17] we therefore keep the binding energies of these SACs calculated from isolated atoms in our work, which can be used to reflect the binding strengths.

Table S4 The calculated binding energies of TM atoms in their corresponding molecular precursors ($E_{b,\text{precursor}}$) and SACs ($E_{b,\text{SAC}}$), as well as the energy difference between $E_{b,\text{precursor}}$ and $E_{b,\text{SAC}}$ ($E_{\Delta b}$).

Precursor	$E_{b,\text{precursor}}$	**SAC**	$E_{b,\text{SAC}}$	$E_{\Delta b}$
$Pd(hfac)_2$[16]	-0.99	Pd@P↓-In_2Se_3	-1.69	-0.70
		Pd@P↑-In_2Se_3	-1.14	-0.15
$Rh(acac)_3$[18]	-0.01	Rh@P↓-In_2Se_3	-4.09	-4.08
		Rh@P↑-In_2Se_3	-2.36	-2.35
$Ni(acac)_2$[19]	-1.42	Ni@P↓-In_2Se_3	-2.72	-1.30
		Ni@P↑-In_2Se_3	-1.97	-0.55

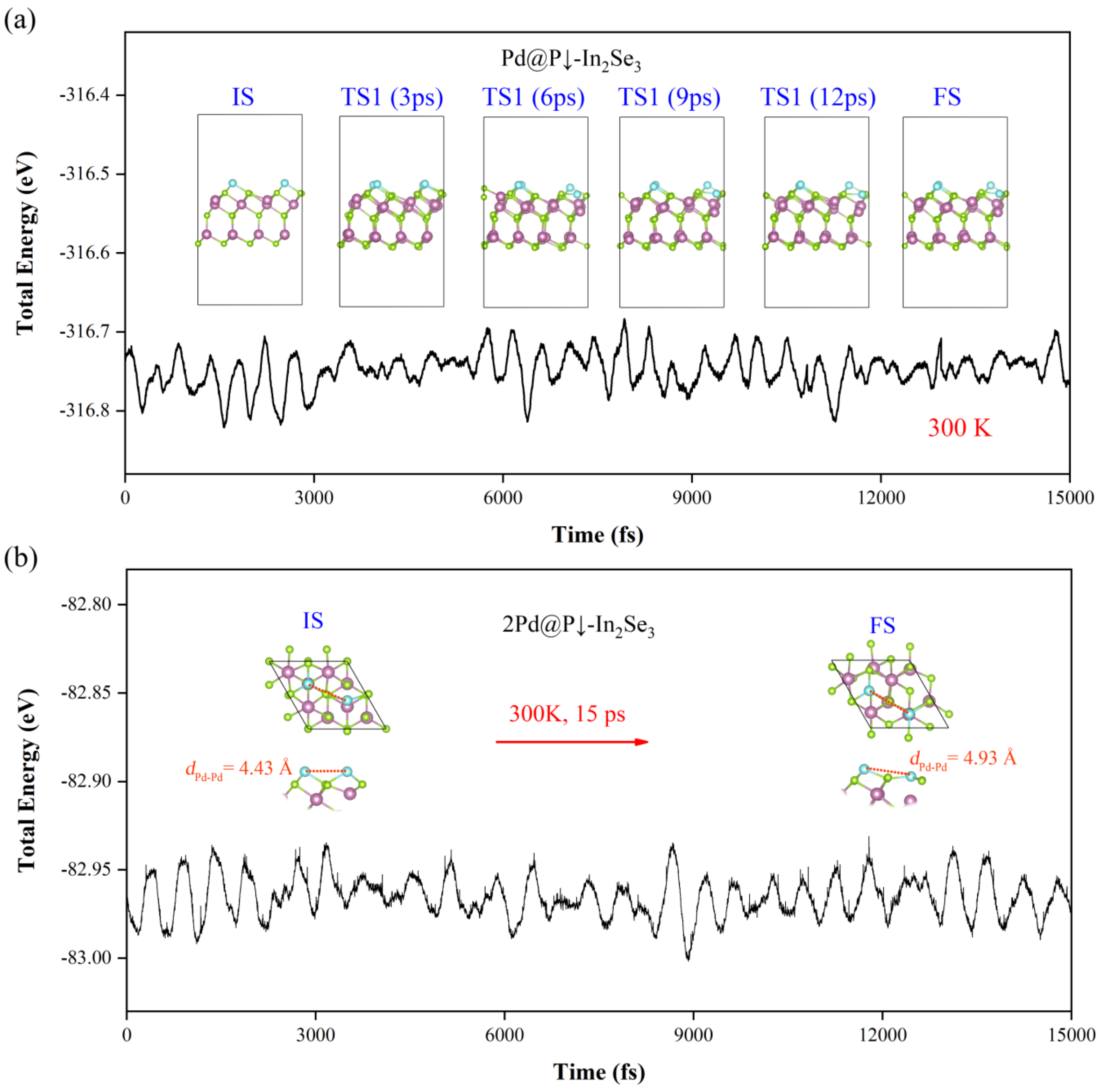

Figure S11 *Ab initio* molecular dynamics (AIMD) results of (a) the Pd@P↓-In_2Se_3 2×2 super cell, where the energy fluctuation is from the thermal disturbance induced by the temperature; (b) 2Pd@P↓-In_2Se_3 unit cell for 15 ps with a time step of 1 fs at 300 K. The insert shows the configuration snapshots of the initial state (IS) and the final state (FS).

The clustering energy

The clustering tendency of Pd atoms on the surface is estimated by the clustering energy ($E_{cluster}$),[20, 21, 22] which is defined as the difference in binding energies between a single metal atom ($E_{b,sin}$) and the metal dimer ($E_{b,dim}$):

$$E_{cluster} = E_{b,sin} - E_{b,dim} \quad \text{(ES8)}$$

where ($E_{b,sin}$) and ($E_{b,dim}$) are defined as:

$$E_{b,sin} = E_{Pd@In_2Se_3} - E_{In_2Se_3} - E_{Pd-bulk} \quad \text{(ES9)}$$

$$E_{b,dim} = \frac{1}{2}(E_{Pd_2/In_2Se_3} - E_{In_2Se_3} - 2E_{Pd-bulk}) \quad \text{(ES10)}$$

where $E_{Pd-bulk}$ is the chemical potential of the Pd atoms in their bulk phase, and E_{Pd_2/In_2Se_3} represents the total energy of the substrate with a Pd dimer. According to the definitions, negative values of $E_{cluster}$ mean that the metal cluster does not tend to form. The calculated values of $E_{cluster}$ are -0.3 eV and -0.04 eV for Pd@P↓-In_2Se_3 and Pd@P↑-In_2Se_3, respectively. To further analyze the stability of single-atom adsorption, we have performed first-principles finite-temperature molecular dynamics simulations of two dispersedly adsorbed Pd atoms on P↓-In_2Se_3 (2Pd@P↓-In_2Se_3) with a Nose–Hoover thermostat at 300 K. The fluctuations of the temperature and the total energy as a function of the simulation time are given in **Figure S11b**. The two Pd atoms maintain dispersedly adsorbed features for at least 15 ps. The distance between the two Pd atoms stays essentially unchanged. All these results confirm the dynamic stability of the single-atom adsorption at room temperature.

The kinetic Monte Carlo (kMC) simulations

KMC simulation method. The details of kMC method have been described in previous works.[23, 24, 25] In this case, we start with a perfect α-In_2Se_3 monolayer with evenly distributed single metal atoms. The simulation size of the α-In_2Se_3 monolayer is ~400×400 $Å^2$ with the periodic boundary conditions and the density of Pd monomers is ~4% (the same model and method also used for Nb case), as shown in **Figure 1a**. During the annealing process, all Pd atoms (including Pd monomers and Pd atoms in Pd_N (N≤3) clusters) can diffuse to the nearest-neighbour sites on α-In_2Se_3 monolayer. Here for simplicity, we ignore the diffusion of Pd atoms in Pd_N (N>3) clusters because large Pd_N (N>3) clusters are very stable. Three elementary rate processes are emphasized in our KMC model: (1) Diffusion of Pd monomers (D1); (2) Diffusion of Pd atoms in Pd_2 clusters (D2); and (3) Diffusion of Pd atoms in Pd_3 clusters (D3). We denote the activation barriers of three processes by V_{D1}, V_{D2}, and V_{D3}, respectively, and the corresponding rates by R_{D1}, R_{D2}, and R_{D3}, with $R_i = v_0 \exp(-V_i/k_B T)$, where V_i is the activation barrier for process i, k_B is the Boltzmann's constant, and T is the annealing temperature. The attempt frequency is chosen as $v_0 = 2k_B T/h = 4.167 \times 10^{10} T$, in which h is the Planck's constant. All the values of the activation barriers used in our KMC simulations are from the DFT calculations, which are listed in **Table S5**.

KMC simulation results. P↓-In_2Se_3 monolayer with evenly distributed Pd atoms are firstly chosen as the initial configuration to simulate the annealing process (**Figure S12a**). **Figure S12b-12f** show the surface morphologies of Pd@P↓-In_2Se_3 after 100 s annealing process at different temperatures. At the low temperatures of T≤350 K (**Figure S12b**), the diffusion of Pd monomers does not happen, and the surface morphology of the Pd@P↓-In_2Se_3 after annealing keeps the same as the initial case. At the intermediate temperatures of T=390~410K (**Figure S12c** and **S12d**), the Pd monomers start to diffuse on α-In_2Se_3 monolayer, but no Pd_N (N≥2) cluster is formed during the annealing process. When the temperature is further increased to T=470 K (**Figure S12e**), the Pd_N (N≥4) clusters start to form although the majority of Pd adatoms are monomers, which indicates that the single Pd atoms on the α-In_2Se_3 monolayer are not stable from 470 K. At the higher temperature of T=490K (**Figure S12f**), almost all the Pd adatoms form Pd_N (N≥4) clusters. Considering that the Pd@P↓-In_2Se_3 are stable for T≤410K, our kMC simulations clearly show the high stability of Pd@P↓-In_2Se_3 electrocatalyst in real electrocatalytic reaction conditions, which will not agglomerate at room temperature (i. e. 293K) environments for at least 100 seconds.

Table S5 The values of activation barriers used in the KMC simulations, which are obtained from the DFT calculations: binding energies of metal monomers and metal atoms in metal clusters (E_i in eV/atom) and diffusion barriers of metal monomers and metal atoms in metal clusters (V_i in eV/atom).

		$\boldsymbol{E_i}$	$\boldsymbol{V_i}$
**Pd@P↓-In_2Se_3**	Pd monomer	-1.69	1.19
	Pd atoms in Pd_2 cluster	-1.38	0.88
	Pd atoms in Pd_3 cluster	-1.99	1.49
	Pd atoms in Pd_4 cluster	-2.66	2.16
**Pd@P↑-In_2Se_3**	Pd monomer	-1.14	0.84
	Pd atoms in Pd_2 cluster	-1.10	0.80
	Pd atoms in Pd_3 cluster	-1.52	1.22
	Pd atoms in Pd_4 cluster	-1.93	1.63
**Nb@P↓-In_2Se_3**	Nb monomer	-7.32	4.63
	Nb atoms in Pd_2 cluster	-6.48	3.79
	Nb atoms in Pd_3 cluster	-6.94	4.25
	Nb atoms in Pd_4 cluster	-7.46	4.77
**Nb@P↑-In_2Se_3**	Nb monomer	-5.92	4.21
	Nb atoms in Pd_2 cluster	-5.82	4.11
	Nb atoms in Pd_3 cluster	-5.86	4.15
	Nb atoms in Pd_4 cluster	-6.37	4.66

The kMC result for Pd@P↑-In_2Se_3 is similar (**Figure S13**), although the starting temperature to form the Pd cluster is much lower than that of Pd@P↑-In_2Se_3 due to lower diffusion barrier (see **Table S5**), no Pd clusters form at T≤300K for 100 seconds, indicating the stability at the room temperature environments. Since Pd@P↑-In_2Se_3 has the lowest migration barrier of 0.84 eV (see Table 1 in the main manuscript), other ferroelectric SACs should not have the issues of TM clustering at the room temperature. For example, the metal agglomerations on ferroelectric surfaces of Nb@P↑-In_2Se_3 and Nb@P↓-In_2Se_3 will not happen at the even 600K for 100s, see **Figure S14**.

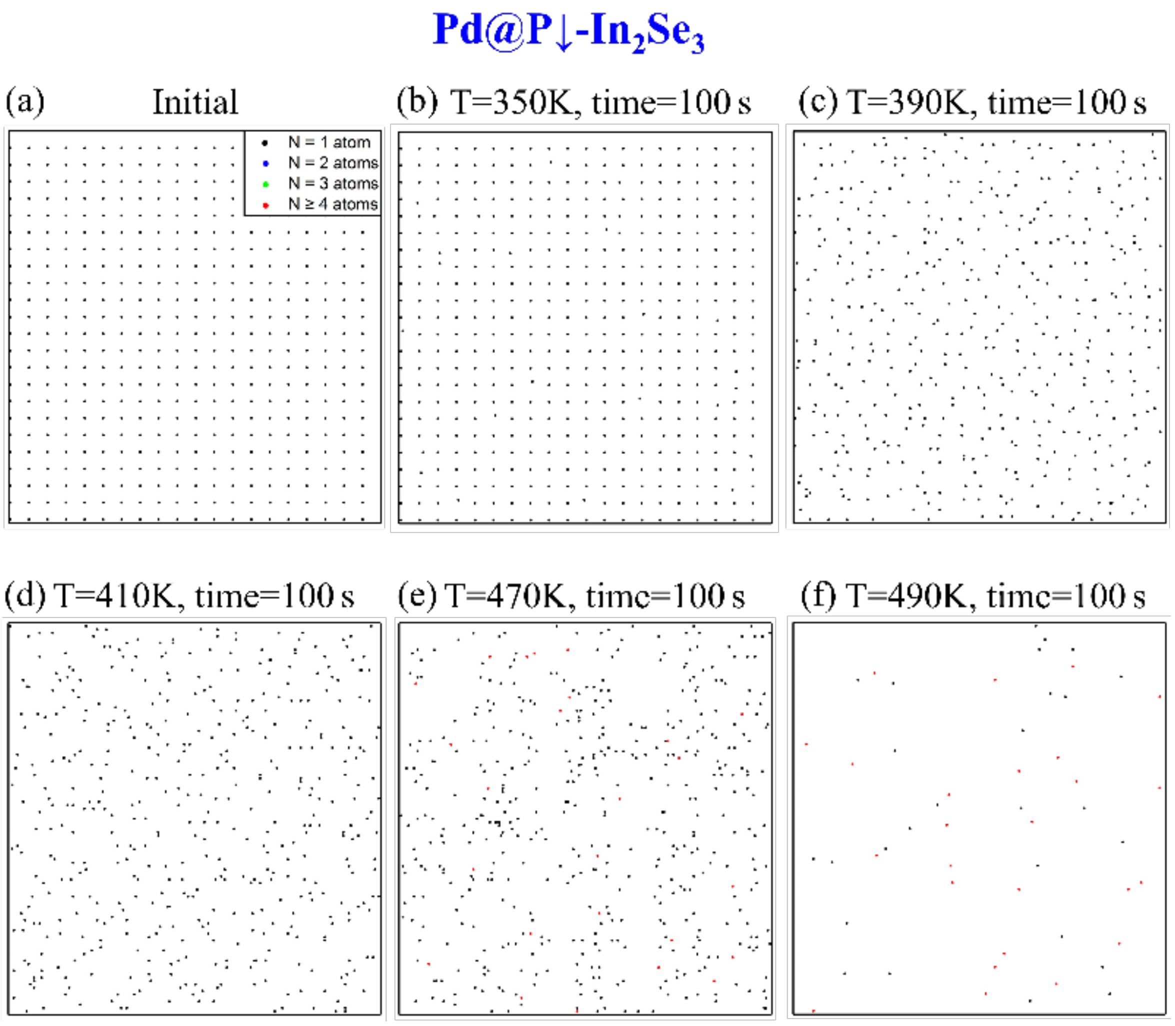

Figure S12 (a) The initial surface morphology of α-In_2Se_3 monolayer with downward (P↓) polarization with evenly distributed Pd atoms (Pd@P↓-In_2Se_3). The surface morphologies of the Pd@P↓-In_2Se_3 electrocatalysts annealed for 100 s at different temperatures: (b) 350 K, (c) 390 K, (d) 410 K, (e) 470 K, and (f) 490 K. Note that no Pd clusters are formed at T≤410 K.

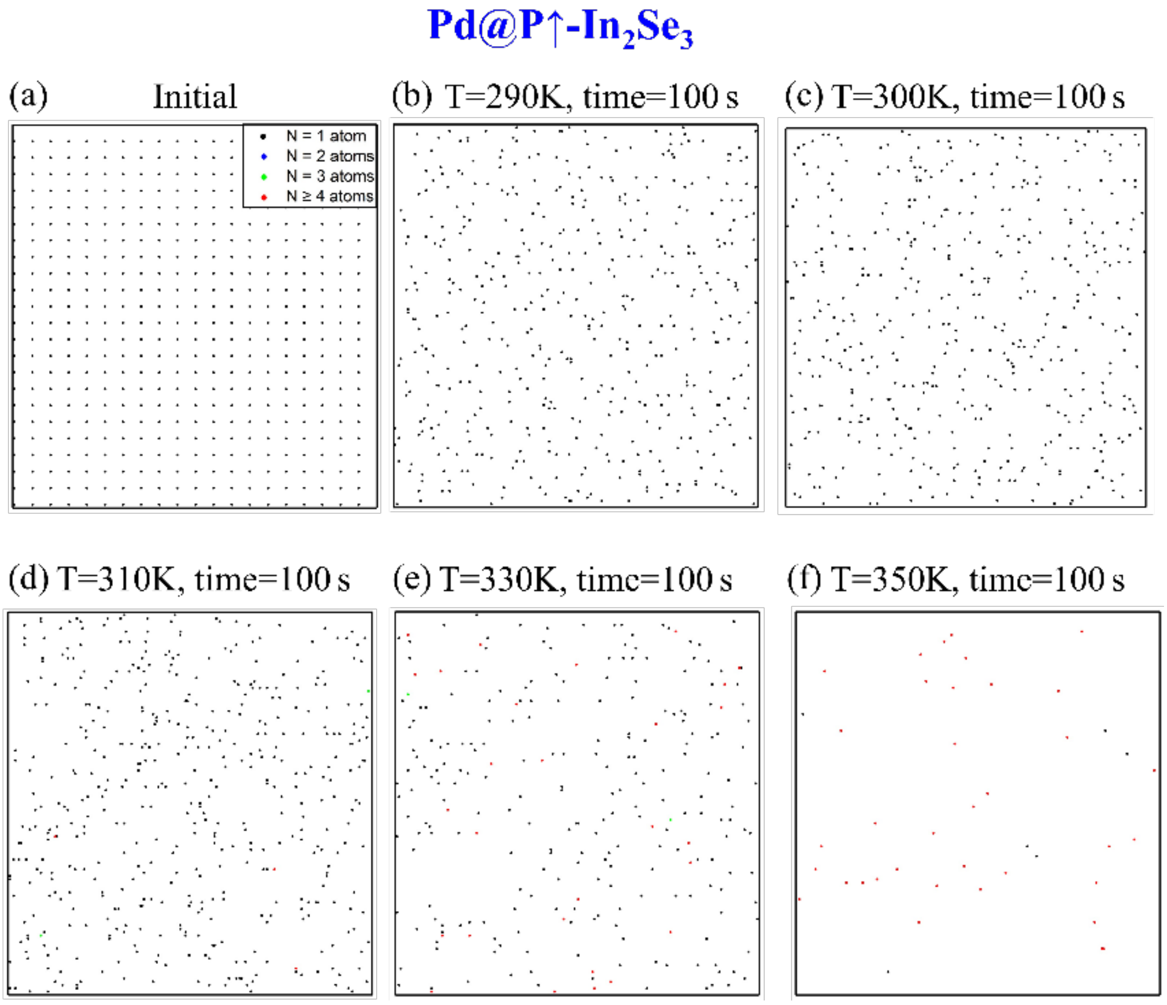

Figure S13 (a) The initial surface morphology of α-In_2Se_3 monolayer with upward (P↑) polarization with evenly distributed Pd atoms (Pd@P↑-In_2Se_3). The surface morphologies of the Pd@P↑-In_2Se_3 electrocatalysts annealed for 100 s at different temperatures: (b) 290 K, (c) 310 K, (d) 330 K, (e) 350 K, and (f) 370 K. No Pd clusters are formed at T≤300 K.

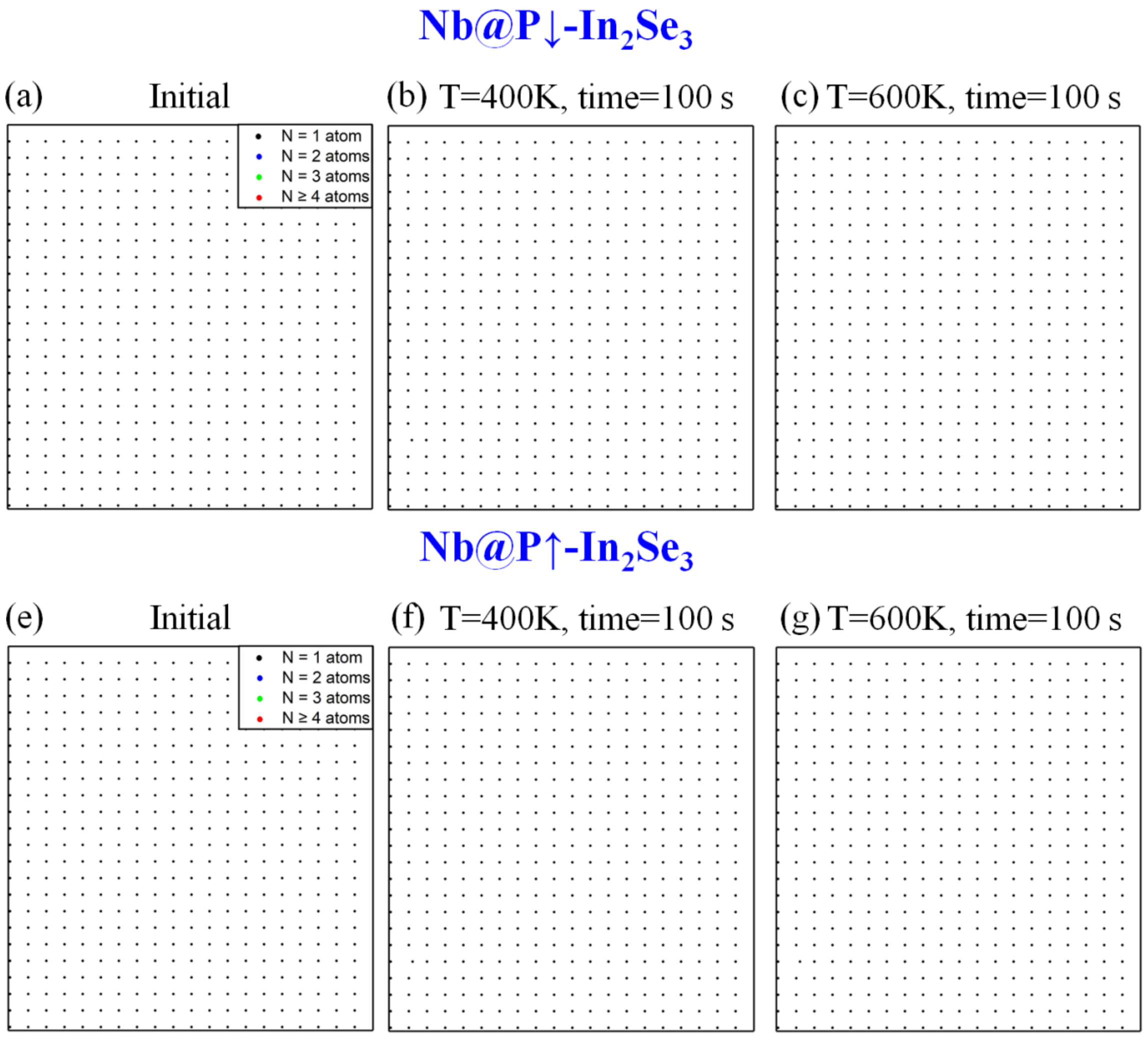

Figure S14 The initial surface morphology of α-In_2Se_3 monolayer with evenly distributed Nb atoms ((a) Nb@P↓-In_2Se_3; (d) Nb@P↑-In_2Se_3). Their surface morphologies of the electrocatalysts annealed for 100 s at different temperatures: (b), (e) 400 K; (c), (f) 600 K. No Nb clusters are formed at T≤600 K

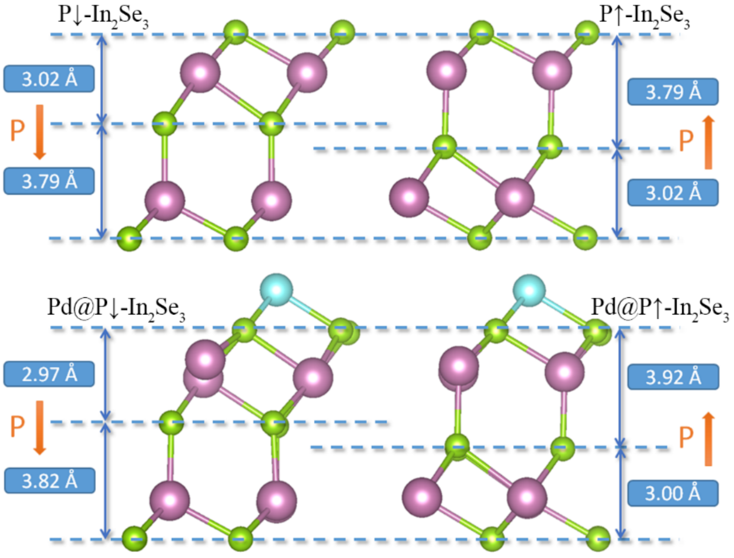

Figure S15 The out-of-plane polarization in bare α-In_2Se_3 monolayer (up) and Pd@In_2Se_3 systems (down).

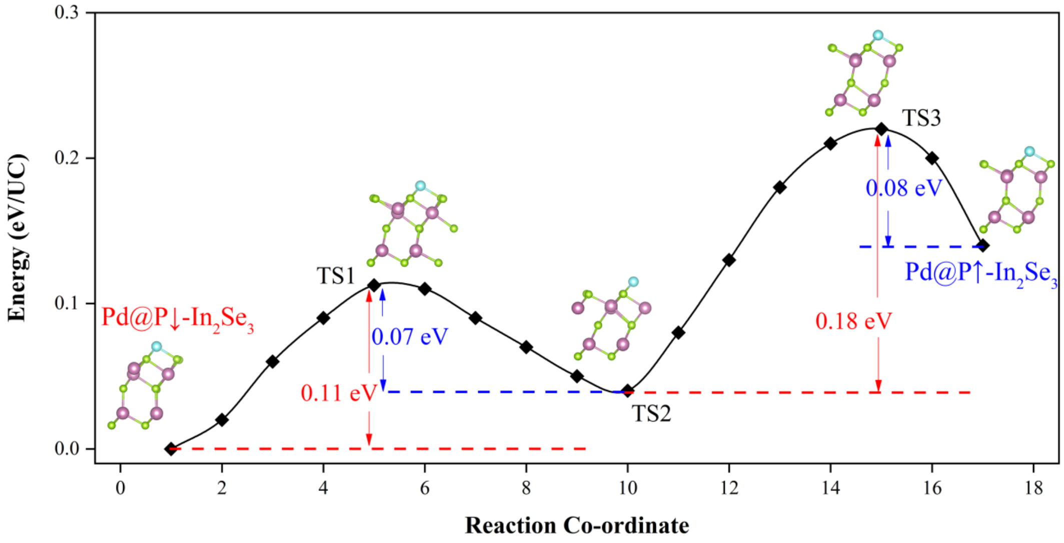

Figure S16 Energy profile of the most effective kinetic pathway to reverse the electric polarization orientation of Pd@In_2Se_3 system. “eV/UC” means “eV per In_2Se_3 unit cell”. For the Pd@In_2Se_3 configuration, there are four In_2Se_3 unit cells. These results are based on the CI-NEB method.

Local metallization in TM@In_2Se_3

Since ferroelectricity generally stems from the offset positive/negative charge centers, the switchability of polarization is closely related with the electronic structures, and the semiconducting or insulating feature is essential for the ferroelectrics.

The catalysts studied here are locally metallized, with the metallic states mainly from the anchored metal atoms and their immediate surrounding area. To prove this point, we have built a large supercell with Pd anchored 6×6 P↓-In_2Se_3 monolayer as shown in **Figure S17** (left). Based on the projected DOS analysis (see the right of **Figure S17**), obviously the metallic density of electronic states near the Fermi level is primarily from Pd and the area near the SAC adsorption site (in the red circle). The other parts (in the blue and green circles) away from the anchor site are semiconducting or insulating. The overall ferroelectricity of the metal anchored In_2Se_3 can thus be well preserved. Therefore, the system will show proper switching behaviors under the electric field.

For Pd@ 6×6 P↑-In_2Se_3, the semiconducting property is reserved since the *d* orbital of Pd is shifted by the polarization. The polarization switching behavior with electric field will remain. For other metal doped In_2Se_3, the ferroelectricity and switching behaviors under electric field can be also reserved due to the same mechanism.

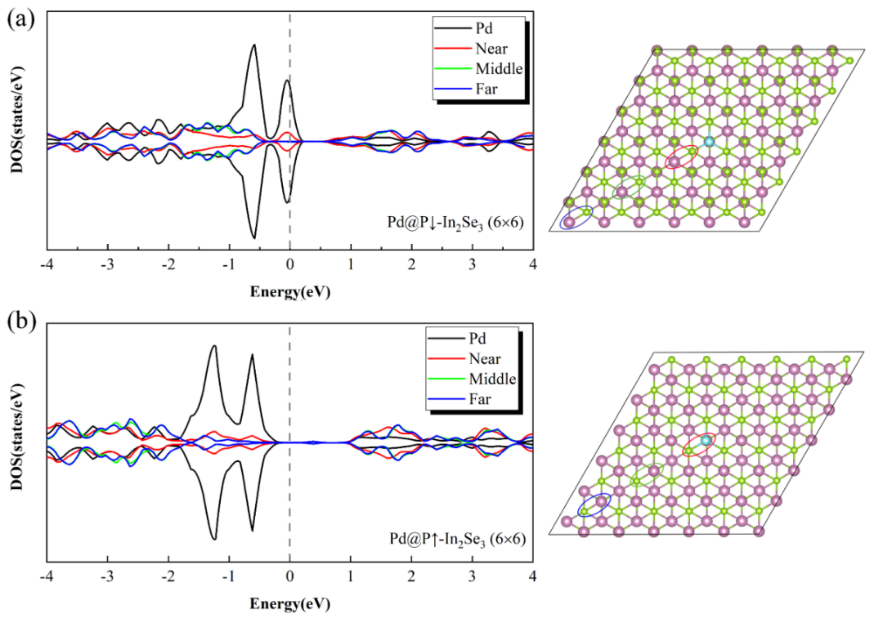

Figure S17 Partial density of states of the Pd SAC, which is constructed by one Pd atom on 6×6 (a) P↓- and (b) P↑-In_2Se_3 supercell. The red, green, and blue lines represent the total density of states of the selected region circled by the red, green and blue lines, which are labeled as "near", "middle" and "far" samples away from the Pd atom.

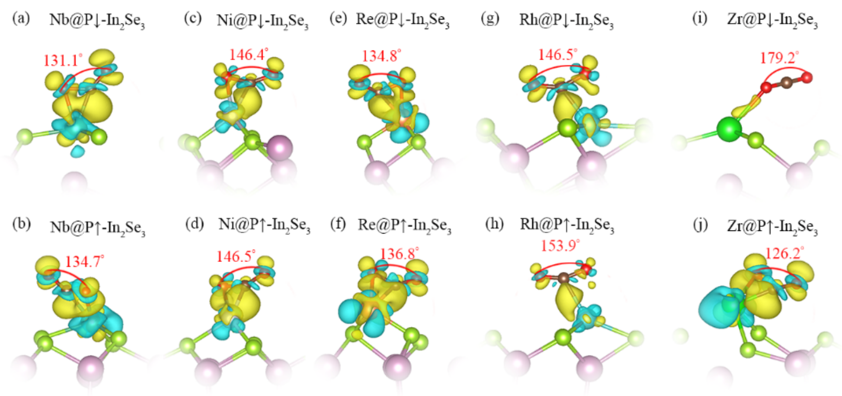

Figure S18 The differential charge densities plots of CO_2 adsorbed on TM@In_2Se_3 ((a), (c), (e), (g) and (i) for TM@P↓-In_2Se_3, and (b), (d), (f), (h) and (j) for TM@P↑-In_2Se_3) (TM=Nb, Ni, Re, Rh and Zr).

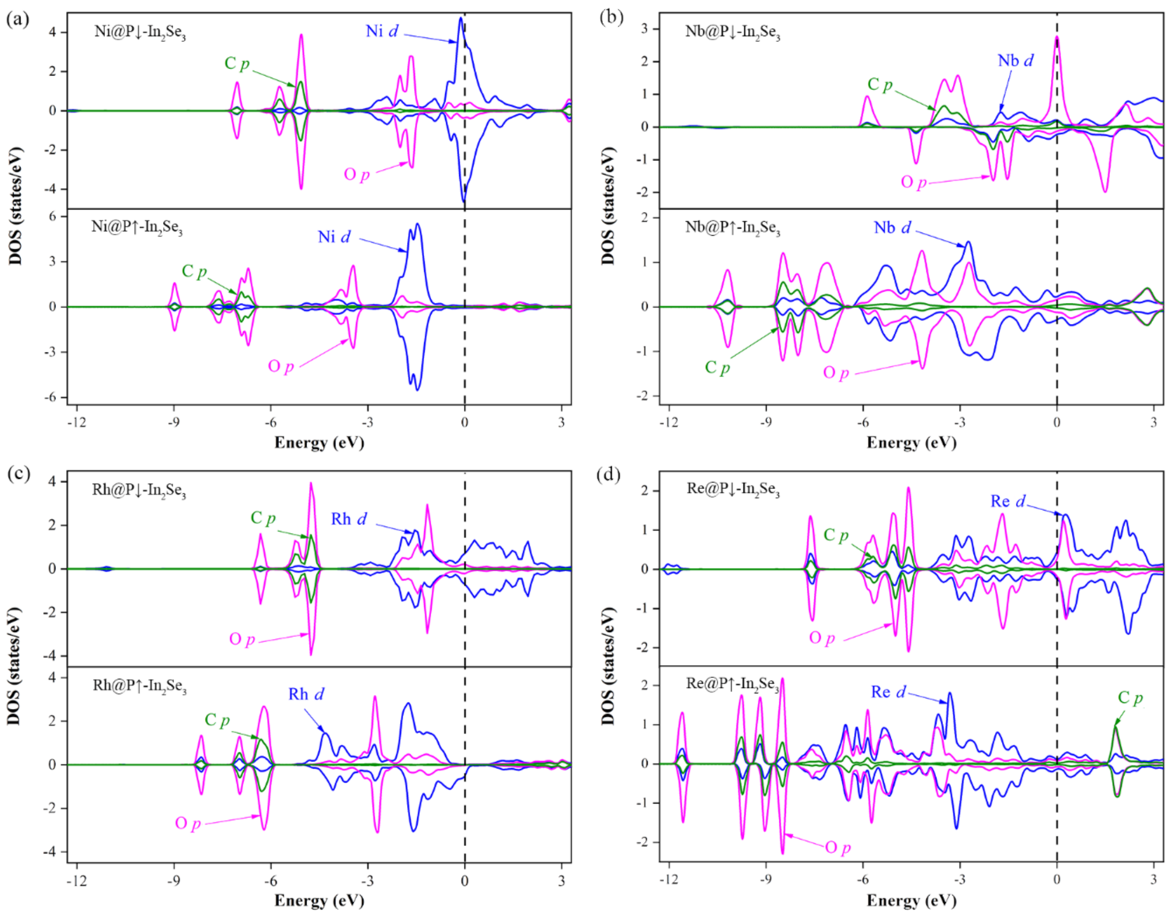

Figure S19 The partial density of states of CO_2 adsorbed on TM@In_2Se_3 (TM= (a) Nb, (b) Ni, (c) Rh, and (d) Re) including TM@P↓-In_2Se_3 and TM@P↑-In_2Se_3 configurations.

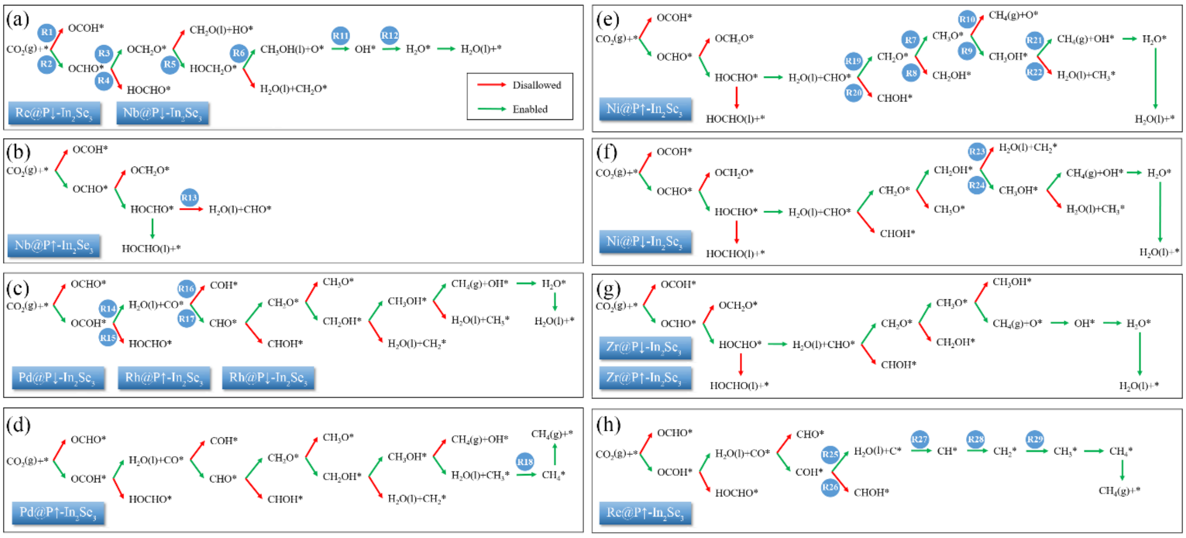

Figure S20 The search process for the minimum energy reaction pathways of the CO_2 reduction reactions on (a) Nb@P↓-In_2Se_3 and Re@P↓-In_2Se_3, (b) Nb@P↑-In_2Se_3, (c) Pd@P↓-In_2Se_3 and Rh@In_2Se_3, (d) Pd@P↑-In_2Se_3, (e) Ni@P↑-In_2Se_3, (f) Ni@P↓-In_2Se_3, (g) Zr@In_2Se_3, and (h) Re@P↑-In_2Se_3 systems. The red arrows denote disallowed reaction paths, while the green arrows stand for the enabled ones.

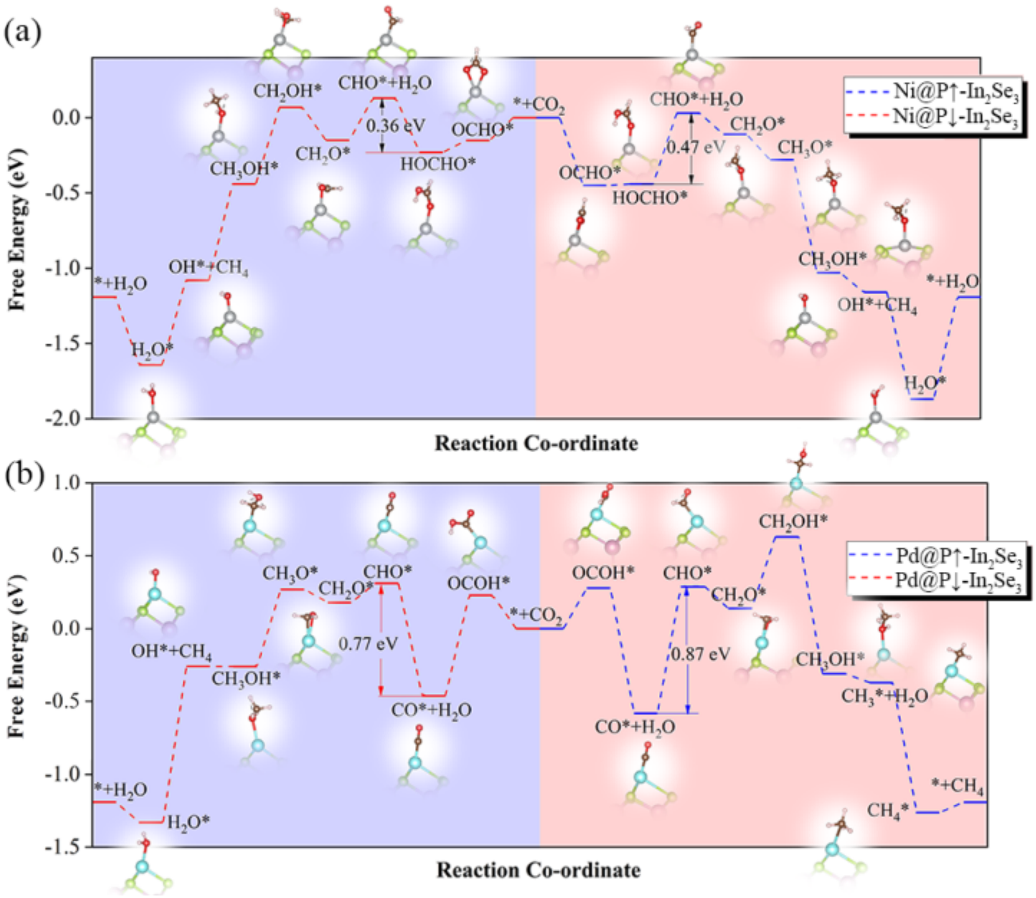

Figure S21 The free-energy profile for CO_2 electrochemical reduction reactions along the minimum energy path at 0 V (*vs.* RHE) on (a) Ni@In_2Se_3, and (b) Pd@In_2Se_3. The insets show the optimized configurations of the intermediates.

Local *vs.* whole ferroelectric switching

To check if the local or whole ferroelectric switching is energetically preferred, we have built a supercell with HOCHO* adsorbed Zr@6×6 P↑-In_2Se_3 (**Figure S22a**) and calculated the energy differences of the structure with ferroelectric switching at only small (**Figure S22b**) or large (**Figure S22c**) local area around Zr anchored site and (**Figure S22d**) entire lattice.

The calculated results indicate that the local ferroelectric switching is indeed energetically prohibitive, and the cases (b) & (c) have much higher energies than that of (d). However, the ferroelectric switching of the entire lattice is not difficult, even when the single metal atoms are fairly apart from each other (24.41 Å): the energy difference is 0.4 eV/6×6cell or 0.045 eV/2×2cell, it is comparable to the data shown in the manuscript. Therefore, we conclude that the ferroelectric switching can readily occur throughout the entire lattice due to the energetic preference when HOCHO* exists.

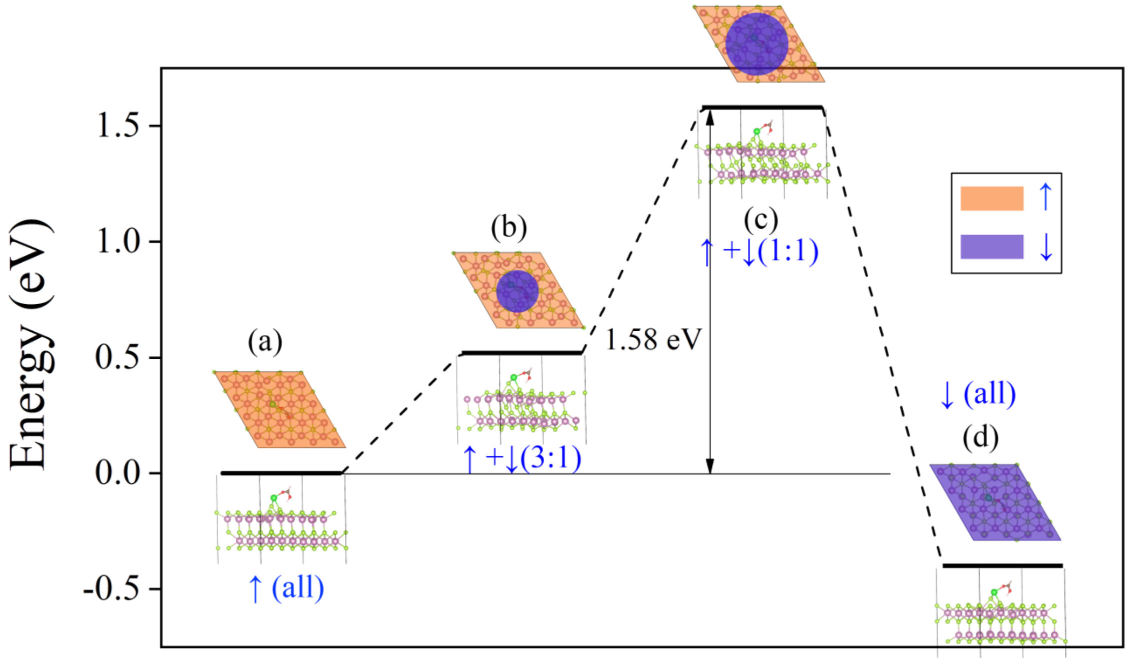

Figure S22 Calculated energies of Zr@In_2Se_3(6×6) with the HCOOH adsorption. Orange and purple areas stand for the up and down polarization areas, respectively. (a) The polarization of In_2Se_3 is pointing upwards; (b) polarization flip at the small area of Zr anchored site (25%); (c) polarization flip at the larger area of Zr anchored site (50%); (d) polarization flip at the entire lattice (100%).

Table S6 Theoretical Limiting Potentials (U_l, V *vs.* RHE) and Experimental Onset Potentials (U_{onset}, V *vs.* RHE) of Various CO_2 RR Catalysts for CH_4 Production.

Theory	U_l	Experiments	U_{onset}
Cu(211)[26]	−0.80	Cu foil[27]	−0.75
Ru@dv-Gr, Os@dv-Gr[28]	−0.52	Cu_2O-derivedCu[29]	−0.65
$Mo_2C(100)$[30]	−0.56	Mo_2C[30]	−0.55
WC(0001)[31]	−1.00	Ni_5Ga_3[32]	−0.50
LiFeAs[33]	−0.55	Bimetallic Cu−Pd[34]	−0.40
Co@Cu[35]	−0.87	Graphene quantum dots[36]	−0.48

Binding energy

The binding energy for the added TM single atom ($E_{b\text{-}TM}$) onto the substrate is calculated by the following equation:[37, 38]

$$E_{b-TM} = E_{TM-sub} - E_{sub} - E_{TM} \quad \text{(ES11)}$$

where E_{sub} and E_{TM-sub} are the total energies of the substrate without and with the added TM single atom, respectively. E_{TM} is the total energy of the isolated TM atom. The binding energy for the adsorbed CO_2 molecule ($E_{b\text{-}CO2}$) onto TM@In_2Se_3 (TM=Nb, Ni, Pd, Rh, Re and Zr) is evaluated in the similar way.

Charge density difference

The charge density difference for TM@In_2Se_3 (TM=Nb, Ni, Pd, Rh, Re and Zr) ($\Delta\rho_1$) is calculated by the following equation:

$$\Delta\rho_1 = \rho_{TM-sub} - \rho_{sub} - \rho_{TM} \quad \text{(ES12)}$$

where ρ_{sub} and ρ_{TM-sub} are the charge density of the substrate without and with the added TM single atom, respectively. ρ_{TM} is the charge density of the added TM atom. The charge density difference for the CO_2 molecule adsorbed TM@In_2Se_3 (TM=Nb, Ni, Pd, Rh, Re and Zr) ($\Delta\rho_2$) is evaluated in the similar way.

d band centre

In order to get the individual orbital components, we employed the code named "split-dos.dos" to process the output file of density of states (DOS). The average *d*-band (*d* band center) shifts are calculated for the surface metal atoms for both the total *d* partial DOS and the orbital-resolved *d* partial DOS. The *d* band center (ε_d) is calculated as:[39]

$$\varepsilon_d = \frac{\int n_d(\varepsilon)\varepsilon d\varepsilon}{\int n_d(\varepsilon) d\varepsilon} \quad \text{(ES13)}$$

Where ε is the electronic energy of states, and $n_d(\varepsilon)$ is the electronic density of states.

Reaction free energy change

The reaction free energy change (ΔG) for each elementary step is calculated based on the computational hydrogen electrode (CHE) model proposed by Nørskov and co-workers by the following equation:[40, 41]

$$\Delta G = \Delta E + \Delta E_{ZPE} - T\Delta S + eU + \Delta G_{pH} \quad \text{(ES14)}$$

where ΔE is the reaction energy obtained from DFT calculations. ΔE_{ZPE} and $T\Delta S$ are the contributions of the zeropoint energy and entropy to ΔG, respectively, which are obtained from the vibrational frequency. *T* is the temperature and taken as 298.15 K, and ΔS is the entropy change.

The vibrational frequencies of molecules in the gas phase are taken from the NIST database,[15] and others are calculated with considering solvent effect. **Tables S7-S19** present E_{ZPE} and TS (at 298.15 K) of the free molecules and the adsorbed species along the most favourable reaction pathway for the TM@In_2Se_3 (TM =Nb, Ni, Pd, Re, Rh, and Zr) catalyst including TM-down and TM-up configurations. e and U are the number of electrons transferred and the electrode potential applied, respectively. ΔG_{pH} is the free energy correction of pH, which is calculated as follows:

$$\Delta G_{pH} = k_B T \times ln10 \times pH \quad \text{(ES15)}$$

where k_B is the Boltzmann constant, and pH = 0 is assumed in an acidic medium in this study. The value of limiting potential (U_l) is determined by the potential-determining step (PDS), which has the most positive ΔG (ΔG_{max}), as computed as follows:

$$U_l = \Delta G_{max}/e \tag{ES16}$$

Table S7 Zero-pint energy correction (E_{ZPE}) and entropy contribution (TS, T=298.15 K) of molecules, which are taken from the NIST database.[15]

Species	TS (eV)	E_{ZPE} (eV)
H_2 (g)	0.40	0.28
CH_4 (g)	0.46	1.20
CO_2 (g)	0.66	0.31
CO (g)	0.61	0.14
H_2O (l)	0.38	0.59
CH_2O (l)	0.40	0.73
CH_3OH (l)	0.47	1.37
HOCHO (l)	0.40	0.90

Table S8 Zero-pint energy correction (E_{ZPE}), entropy contribution (TS, T=298.15 K), total energy (E), and the Gibbs free energy (G) of molecules and adsorbates along the reaction pathway on Nb@P↓-In_2Se_3, where * represents the adsorption site.

Species	E (eV)	TS (eV)	E_{ZPE} (eV)	G(eV)
H*	-88.11	0.02	0.15	-87.98
OCOH*	-112.39	0.24	0.62	-112.01
OCHO*	-113.28	0.20	0.62	-112.86
OCH_2O *	-116.74	0.21	0.92	-116.03
$HOCH_2O$*	-120.4	0.24	1.24	-119.4
O*	-93.66	0.08	0.07	-93.67
OH*	-97.37	0.13	0.33	-97.17
H_2O*	-100.45	0.17	0.64	-99.98

Table S9 Zero-pint energy correction (E_{ZPE}), entropy contribution (TS, T=298.15 K), total energy (E), and the Gibbs free energy (G) of adsorbates along the reaction pathway on Nb@P↑-In_2Se_3, where * represents the adsorption site.

Species	E (eV)	TS (eV)	E_{ZPE} (eV)	G(eV)
H*	-87.03	0.01	0.17	-86.87
OCOH*	-111.51	0.23	0.62	-111.12
OCHO*	-112.43	0.20	0.63	-112.00
HOCHO*	-115.52	0.27	0.92	-114.87
CHO*	-103.07	0.16	0.47	-102.76

Table S10 Zero-pint energy correction (E_{ZPE}), entropy contribution (TS, T=298.15 K), total energy (E), and the Gibbs free energy (G) of adsorbates along the reaction pathway on Ni@P↓-In_2Se_3, where * represents the adsorption site.

Species	E (eV)	TS (eV)	E_{ZPE} (eV)	G(eV)
H*	-83.17	0.06	0.14	-83.09
OCOH*	-107.03	0.27	0.61	-106.69
OCHO*	-107.37	0.23	0.61	-106.99
HOCHO*	-111.12	0.33	0.93	-110.52
CHO*	-99.53	0.21	0.46	-99.28
CH_2O*	-103.57	0.21	0.77	-103.01
CH_2OH*	-107.11	0.23	1.10	-106.24
CH_3OH *	-111.31	0.30	1.41	-110.20
OH*	-91.17	0.17	0.33	-91.01
H_2O*	-95.45	0.22	0.65	-95.02

Table S11 Zero-pint energy correction (E_{ZPE}), entropy contribution (TS, T=298.15 K), total energy (E), and the Gibbs free energy (G) of adsorbates along the reaction pathway on Ni@P↑-In_2Se_3, where * represents the adsorption site.

Species	E (eV)	TS (eV)	E_{ZPE} (eV)	G(eV)
H*	-82.49	0.03	0.16	-82.36
OCOH*	-106.27	0.29	0.60	-105.96
OCHO*	-106.72	0.31	0.60	-106.43
HOCHO*	-110.48	0.31	0.92	-109.87
CHO*	-98.75	0.22	0.45	-98.52
CH_2O*	-102.62	0.25	0.76	-102.11
CH_3O*	-106.50	0.30	1.07	-105.73
CH_3OH *	-111.08	0.26	1.41	-109.93
OH*	-90.41	0.16	0.34	-90.23
H_2O*	-94.83	0.20	0.64	-94.39

Table S12 Zero-pint energy correction (E_{ZPE}), entropy contribution (TS, T=298.15 K), total energy (E), and the Gibbs free energy (G) of adsorbates along the reaction pathway on Pd@P↓-In_2Se_3, where * represents the adsorption site.

Species	E (eV)	TS (eV)	E_{ZPE} (eV)	G(eV)
H*	-82.97	0.03	0.16	-82.84
OCOH*	-107.02	0.28	0.61	-106.69
OCHO*	-106.99	0.23	0.61	-106.61
CO*	-96.52	0.17	0.19	-96.50
CHO*	-99.47	0.18	0.47	-99.18
CH_2O*	-103.24	0.27	0.75	-102.76
CH_2OH*	-107.00	0.22	1.10	-106.12
CH_3OH *	-111.17	0.33	1.40	-110.10
OH*	-90.47	0.13	0.33	-90.27
H_2O*	-95.25	0.19	0.65	-94.79

Table S13 Zero-pint energy correction (E_{ZPE}), entropy contribution (TS, T=298.15 K), total energy (E), and the Gibbs free energy (G) of molecules and adsorbates along the reaction pathway on Pd@P↑-In_2Se_3, where * represents the adsorption site.

Species	E (eV)	TS (eV)	E_{ZPE} (eV)	G(eV)
H*	-82.51	0.02	0.17	-82.36
OCOH*	-106.4	0.30	0.61	-106.09
OCHO*	-106.28	0.22	0.62	-105.88
CO*	-96.1	0.16	0.19	-96.07
CHO*	-98.89	0.22	0.46	-98.65
CH_2O*	-102.8	0.22	0.77	-102.25
CH_2OH*	-106.07	0.22	1.08	-105.21
CH_3OH *	-110.69	0.31	1.40	-109.6
CH_3*	-99.53	0.18	0.93	-98.78
CH_4*	-104.02	0.30	1.20	-103.12

Table S14 Zero-pint energy correction (E_{ZPE}), entropy contribution (TS, T=298.15 K), total energy (E), and the Gibbs free energy (G) of molecules and adsorbates along the reaction pathway on Re@P↓-In_2Se_3, where * represents the adsorption site.

Species	E (eV)	TS (eV)	E_{ZPE} (eV)	G(eV)
H*	-88.42	0.02	0.18	-88.26
OCOH*	-111.69	0.24	0.62	-111.31
OCHO*	-112.38	0.22	0.61	-111.99
OCH_2O *	-115.87	0.20	0.93	-115.14
$HOCH_2O$*	-119.82	0.25	1.25	-118.82
O*	-93.34	0.10	0.06	-93.38
OH*	-96.71	0.11	0.34	-96.48
H_2O*	-99.74	0.18	0.64	-99.28

Table S15 Zero-pint energy correction (E_{ZPE}), entropy contribution (TS, T=298.15 K), total energy (E), and the Gibbs free energy (G) of molecules and adsorbates along the reaction pathway on Re@P↑-In_2Se_3, where * represents the adsorption site.

Species	E (eV)	TS (eV)	E_{ZPE} (eV)	G(eV)
H*	-86.20	0.02	0.18	-86.04
OCOH*	-111.15	0.22	0.61	-110.76
OCHO*	-110.94	0.22	0.61	-110.55
CO*	-99.72	0.17	0.20	-99.69
COH*	-103.06	0.15	0.49	-102.72
C*	-91.00	0.07	0.08	-90.99
CH*	-95.40	0.07	0.35	-95.12
CH_2*	-99.82	0.12	0.60	-99.34
CH_3*	-103.17	0.16	0.92	-102.41
CH_4*	-107.33	0.21	1.20	-106.34

Table S16 Zero-pint energy correction (E_{ZPE}), entropy contribution (TS, T=298.15 K), total energy (E), and the Gibbs free energy (G) of molecules and adsorbates along the reaction pathway on Rh@P↓-In_2Se_3, where * represents the adsorption site.

Species	E (eV)	TS (eV)	E_{ZPE} (eV)	G(eV)
H*	-84.91	0.03	0.15	-84.79
OCOH*	-109.14	0.21	0.61	-108.74
OCHO*	-108.76	0.27	0.60	-108.43
CO*	-98.35	0.18	0.20	-98.33
CHO*	-101.65	0.17	0.47	-101.35
CH_2O*	-105.06	0.19	0.78	-104.47
CH_2OH*	-109.11	0.20	1.11	-108.20
CH_3OH *	-113.10	0.29	1.41	-111.98
OH*	-92.52	0.10	0.35	-92.27
H_2O*	-97.15	0.20	0.64	-96.71

Table S17 Zero-pint energy correction (E_{ZPE}), entropy contribution (TS, T=298.15 K), total energy (E), and the Gibbs free energy (G) of molecules and adsorbates along the reaction pathway on Rh@P↑-In_2Se_3, where * represents the adsorption site.

Species	E (eV)	TS (eV)	E_{ZPE} (eV)	G(eV)
H*	-83.78	0.01	0.21	-83.58
OCOH*	-107.86	0.24	0.62	-107.48
OCHO*	-107.81	0.20	0.62	-107.39
CO*	-96.90	0.18	0.19	-96.89
CHO*	-100.46	0.18	0.48	-100.16
CH_2O*	-103.93	0.20	0.78	-103.35
CH_2OH*	-108.12	0.19	1.11	-107.20
CH_3OH *	-111.88	0.34	1.40	-110.82
OH*	-91.12	0.11	0.34	-90.89
H_2O*	-95.77	0.21	0.64	-95.34

Table S18 Zero-pint energy correction (E_{ZPE}), entropy contribution (TS, T=298.15 K), total energy (E), and the Gibbs free energy (G) of molecules and adsorbates along the reaction pathway on Zr@P↓-In_2Se_3, where * represents the adsorption site.

Species	E (eV)	TS (eV)	E_{ZPE} (eV)	G(eV)
HOCHO*	-116.16	0.24	0.89	-115.51
CHO*	-104.28	0.18	0.44	-104.02
CH_2O*	-109.15	0.20	0.76	-108.59
CH_3O*	-113.36	0.22	1.09	-112.49
O*	-93.40	0.10	0.06	-93.44
OH*	-97.02	0.22	0.30	-96.94
H_2O*	-100.03	0.22	0.63	-99.62

Table S19 Zero-pint energy correction (E_{ZPE}), entropy contribution (TS, T=298.15 K), total energy (E), and the Gibbs free energy (G) of molecules and adsorbates along the reaction pathway on Zr@P↑-In_2Se_3, where * represents the adsorption site.

Species	E (eV)	TS (eV)	E_{ZPE} (eV)	G(eV)
H*	-86.18	0.01	0.16	-86.03
OCOH*	-110.20	0.30	0.55	-109.95
OCHO*	-112.09	0.21	0.62	-111.68
HOCHO*	-116.19	0.26	0.90	-115.55
CHO*	-102.63	0.17	0.46	-102.34
CH_2O*	-107.92	0.18	0.77	-107.33
CH_3O*	-112.86	0.25	1.09	-112.02
O*	-91.89	0.09	0.06	-91.92
OH*	-95.73	0.14	0.32	-95.55
H_2O*	-99.52	0.17	0.65	-99.04

Elementary steps in CO_2RR and HER

All the hydrogenation reactions (R1~R29) considered in the search process for the minimum energy reaction pathways of the CO_2 reduction reactions (see **Figure S21**) can be written as:

$$* + CO_2(g) + H^+ + e^- \rightarrow OCOH* \quad (R1)$$

$$* + CO_2(g) + H^+ + e^- \rightarrow OCHO* \quad (R2)$$

$$OCHO* + H^+ + e^- \rightarrow OCH_2O* \quad (R3)$$

$$OCHO* + H^+ + e^- \rightarrow HOCHO* \quad (R4)$$

$$OCH_2O* + H^+ + e^- \rightarrow HOCH_2O* \quad (R5)$$

$$HOCH_2O* + H^+ + e^- \rightarrow CH_3OH(l) + O* \quad (R6)$$

$$CH_2O* + H^+ + e^- \rightarrow CH_3O* \quad (R7)$$

$$CH_2O* + H^+ + e^- \rightarrow CH_2OH* \quad (R8)$$

$$CH_3O* + H^+ + e^- \rightarrow CH_3OH* \quad (R9)$$

$$CH_3O* + H^+ + e^- \rightarrow CH_4(g) + O* \quad (R10)$$

$$O* + H^+ + e^- \rightarrow OH* \quad (R11)$$

$$OH* + H^+ + e^- \rightarrow H_2O* \quad (R12)$$

$$HOCHO* + H^+ + e^- \rightarrow H_2O(l) + CHO* \quad (R13)$$

$$OCOH* + H^+ + e^- \rightarrow H_2O(l) + CO* \quad (R14)$$

$$OCOH* + H^+ + e^- \rightarrow HOCHO* \quad (R15)$$

$$CO* + H^+ + e^- \rightarrow COH* \quad (R16)$$

$$CO* + H^+ + e^- \rightarrow CHO* \quad (R17)$$

$$CH_3* + H^+ + e^- \rightarrow CH_4* \quad (R18)$$

$$CHO* + H^+ + e^- \rightarrow CH_2O* \quad (R19)$$

$$CHO* + H^+ + e^- \rightarrow CHOH* \quad (R20)$$

$$CH_3OH^* + H^+ + e^- \rightarrow CH_4(g) + OH^* \tag{R21}$$

$$CH_3OH^* + H^+ + e^- \rightarrow H_2O(l) + CH_3^* \tag{R22}$$

$$CH_2OH^* + H^+ + e^- \rightarrow H_2O(l) + CH_2^* \tag{R23}$$

$$CH_2OH^* + H^+ + e^- \rightarrow CH_3OH^* \tag{R24}$$

$$COH^* + H^+ + e^- \rightarrow H_2O(l) + C^* \tag{R25}$$

$$COH^* + H^+ + e^- \rightarrow CHOH^* \tag{R26}$$

$$C^* + H^+ + e^- \rightarrow CH^* \tag{R27}$$

$$CH^* + H^+ + e^- \rightarrow CH_2^* \tag{R28}$$

$$CH_2^* + H^+ + e^- \rightarrow CH_3^* \tag{R29}$$

The hydrogenation reactions (R30~R31) for HER can be written as:

$$* + H^+ + e^- \rightarrow H^* \tag{R30}$$

$$H^* + H^+ + e^- \rightarrow *+H_2(g) \tag{R31}$$

Therefore, when $U = 0$ V and pH = 0, ΔG for each elementary step (R1-R31) can be rewritten as:

$$\Delta G_{R1} = G_{OCOH*} - \frac{1}{2}G_{H_2} - G_* - G_{CO_2} \tag{ES17}$$

$$\Delta G_{R2} = G_{OCHO*} - \frac{1}{2}G_{H_2} - G_* - G_{CO_2} \tag{ES18}$$

$$\Delta G_{R3} = G_{OCH_2O*} - \frac{1}{2}G_{H_2} - G_{OCHO*} \tag{ES19}$$

$$\Delta G_{R4} = G_{HOCHO*} - \frac{1}{2}G_{H_2} - G_{OCHO*} \tag{ES20}$$

$$\Delta G_{R5} = G_{HOCH_2O*} - \frac{1}{2}G_{H_2} - G_{OCH_2O*} \tag{ES21}$$

$$\Delta G_{R6} = G_{CH_3OH} + G_{O*} - \frac{1}{2}G_{H_2} - G_{HOCH_2O*} \tag{ES22}$$

$$\Delta G_{R7} = G_{CH_3O*} - \frac{1}{2}G_{H_2} - G_{CH_2O*} \tag{ES23}$$

$$\Delta G_{R8} = G_{CH_2OH*} - \frac{1}{2} G_{H_2} - G_{CH_2O*} \tag{ES24}$$

$$\Delta G_{R9} = G_{CH_3OH*} - \frac{1}{2} G_{H_2} - G_{CH_3O*} \tag{ES25}$$

$$\Delta G_{R10} = G_{CH_4} + G_{O*} - \frac{1}{2} G_{H_2} - G_{CH_3O*} \tag{ES26}$$

$$\Delta G_{R11} = G_{OH*} - \frac{1}{2} G_{H_2} - G_{O*} \tag{ES27}$$

$$\Delta G_{R12} = G_{H_2O*} - \frac{1}{2} G_{H_2} - G_{OH*} \tag{ES28}$$

$$\Delta G_{R13} = G_{H_2O} + G_{CHO*} - \frac{1}{2} G_{H_2} - G_{HOCHO*} \tag{ES29}$$

$$\Delta G_{R14} = G_{H_2O} + G_{CO*} - \frac{1}{2} G_{H_2} - G_{OCOH*} \tag{ES30}$$

$$\Delta G_{R15} = G_{HOCHO*} - \frac{1}{2} G_{H_2} - G_{OCOH*} \tag{ES31}$$

$$\Delta G_{R16} = G_{COH*} - \frac{1}{2} G_{H_2} - G_{CO*} \tag{ES32}$$

$$\Delta G_{R17} = G_{CHO*} - \frac{1}{2} G_{H_2} - G_{CO*} \tag{ES33}$$

$$\Delta G_{R18} = G_{CH_4*} - \frac{1}{2} G_{H_2} - G_{CH_3*} \tag{ES34}$$

$$\Delta G_{R19} = G_{CH_2O*} - \frac{1}{2} G_{H_2} - G_{CHO*} \tag{ES35}$$

$$\Delta G_{R20} = G_{CHOH*} - \frac{1}{2} G_{H_2} - G_{CHO*} \tag{ES36}$$

$$\Delta G_{R21} = G_{CH_4} + G_{OH*} - \frac{1}{2} G_{H_2} - G_{CH_3OH*} \tag{ES37}$$

$$\Delta G_{R22} = G_{H_2O} + G_{CH_3*} - \frac{1}{2} G_{H_2} - G_{CH_3OH*} \tag{ES38}$$

$$\Delta G_{R23} = G_{H_2O} + G_{CH_2*} - \frac{1}{2} G_{H_2} - G_{CH_2OH*} \tag{ES39}$$

$$\Delta G_{R24} = G_{CH_3OH*} - \frac{1}{2} G_{H_2} - G_{CH_2OH*} \tag{ES40}$$

$$\Delta G_{R25} = G_{H_2O} + G_{C*} - \frac{1}{2} G_{H_2} - G_{COH*} \tag{ES41}$$

$$\Delta G_{R26} = G_{CHOH*} - \frac{1}{2} G_{H_2} - G_{COH*} \tag{ES42}$$

$$\Delta G_{R27} = G_{CH*} - \frac{1}{2}G_{H_2} - G_{C*} \quad \text{(ES43)}$$

$$\Delta G_{R28} = G_{CH_2*} - \frac{1}{2}G_{H_2} - G_{CH*} \quad \text{(ES44)}$$

$$\Delta G_{R29} = G_{CH_3*} - \frac{1}{2}G_{H_2} - G_{CH_2*} \quad \text{(ES45)}$$

$$\Delta G_{R30} = G_{H*} - \frac{1}{2}G_{H_2} - G_{*} \quad \text{(ES46)}$$

$$\Delta G_{R31} = G_{*} + \frac{1}{2}G_{H_2} - G_{H*} \quad \text{(ES47)}$$

The Gibbs free energy (G) of the adsorbates on the TM@In_2Se_3 catalysts along the minimum energy reaction pathway for both CO_2 RR and HER are listed in **Table S8~S19**.

Table S20 The screening results for the 29 kinds of transition metals. **Criteria 1**: the single transition metal atom should be steadily adsorbed on the surface of the In_2Se_3 monolayer, and the favourable adsorption site should not obviously change after the polarization switch. **Criteria 2**: the single transition metal atom should be able to activate CO_2 at least in one polarization state.

Metal	**Criteria 1**	**Criteria 2**		Metal	**Criteria 1**	**Criteria 2**
Sc	Yes	No[5]		Ru	No[2]	--
Ti	Yes	No[5]		Rh	Yes	Yes
V	No[2]	--		Pd	Yes	Yes
Cr	Yes	No[4]		Ag	Yes	No[4]
Mn	No[2]	--		Cd	No[1]	--
Fe	No[2]	--		Hf	Yes	No[5]
Co	No[2]	--		Ta	No[3]	--
Ni	Yes	Yes		W	No[2]	--
Cu	No[1]	--		Re	Yes	Yes
Zn	No[1]	--		Os	No[2]	--
Y	Yes	No[5]		Ir	No[1]	--
Zr	Yes	Yes		Pt	Yes	No[4]
Nb	Yes	Yes		Au	Yes	No[4]
Mo	No[3]	--		Hg	No[1]	--
Tc	No[1]	--				

"Yes" means these metal atoms match the criteria.

"No" means these metal atoms mismatch the criteria. Specifically, "No[1]" means these metal atoms desorb from the In_2Se_3 surface at both up and down polarization phases; "No[2]" means these metal atoms could stably adsorb on the In_2Se_3 surface at only one polarization phase; "No[3]" means the favourable adsorption sites of these metal atoms obviously change after the polarization switch; "No[4]" means these metal atoms can not activate CO_2 molecules at both up and down polarization phases; "No[5]" means these metal atoms can activate CO_2 molecules at only one polarization phases, but the species, composed by the activated CO_2 molecules and the metal atoms, desorb from the In_2Se_3 surface.

"--" means these metal atoms are not considered for the second screening procedure, because they mismatch **Criteria 1**.

The number of catalytic active sites

The simulations were conducted to study the maximum active sites of Pd adatoms on a 4×4 In_2Se_3 supercell. Since the centers of the six-membered ring have been identified as the energetically preferred dopant sites (**Figure 1**), where the metal adsorbents are uniformly dispersed (rather than forming cluster) as the catalytic active sites. Based on the results, the formation energies are calculated to figure out the maximum active sites of SAC, with Pd adatoms evenly distributed 4×4 In_2Se_3 supercell as the representative example. As shown in **Figure S15,** for both Pd@P↑-In_2Se_3 and Pd@P↓-In_2Se_3, when the surface converge is within 25% (namely 4 adatoms are uniformly distributed on the supercell surface), the formation energies are increased (absolute values) and converge to -1.69 and -1.15 eV/Pd respectively. Additional Pd atom on the surface will raise the formation energy due to the reduced distance between the nearest neighbors and the Coulomb repulsion. For example, the fifth Pd adsorbent on 4×4 In_2Se_3 leads to the formation energies of -1.65 and -1.12 eV/Pd atom for two polarization states. We therefore conclude that In_2Se_3 surface can host a large number of catalytic active sites, which are uniformly dispersed with the converge up to 25%.

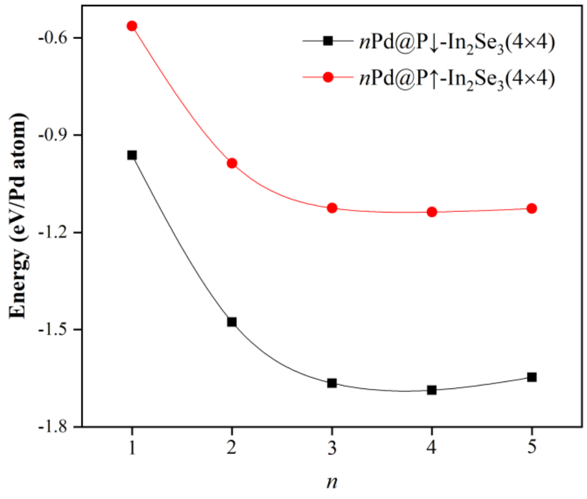

Figure S23 Calculated formation energy versus adatom number (n) for nPd@In_2Se_3 (4×4).

The dependence of catalytic activity on metal atom concentrations

To investigate the dependence of catalytic activity on metal atom concentrations, we have calculated the free-energy profile for CO_2 electrochemical reduction reactions along the minimum energy path at 0 V (vs. RHE) on Pd@In_2Se_3 by using 2×2, 4×4, and 6×6 α-In_2Se_3

supercells. As shown in **Figure S24**, the difference of the free-energy profiles for the CO_2RR between Pd@ In_2Se_3 (4×4) and Pd@In_2Se_3 (6×6) are within 0.05 eV, and the converged free-energy profiles indicate that the catalytic activity is preserved when the doping concentration is in the dilute limit. On the other hand, while the binding strengths of the reaction intermediates for the dilute doping concentration are stronger than those for the high concentration doping case, the rate limited steps for CO_2RR are the same, and the overpotential difference of CO_2RR between the high and low concentrations is less than 0.13 eV. These results confirm that the CO_2RR activities of metal doped In_2Se_3 with high and low concentrations are quite similar, and the doping concentration on metal adsorbed In_2Se_3 has little effect on our main conclusions.

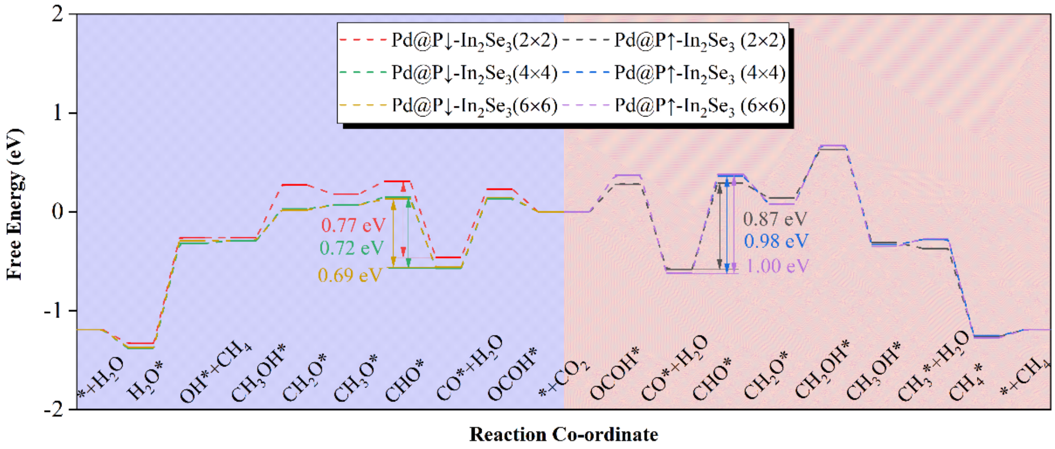

Figure S24 The free-energy profile for CO_2 electrochemical reduction reactions along the minimum energy path at 0 V (vs. RHE) on Pd@In_2Se_3 by using 2×2, 4×4, and 6×6 α-In_2Se_3 supercells.

PBE *vs.* PBE+U

The inclusion of the Hubbard-U term via, e.g., the DFT+U approach, may be more suitable for systems with highly localized orbitals. However, the DFT+U approach also suffers from a strong (linear) dependence of the energetics on the choice of the value of the parameter *U*, and on the choice of the localized projector functions that enter the definition of the *U*-dependent energy term. For example, the reduction energy (ΔH) of $CeO_2 \rightarrow Ce_2O_3$ process can vary between −5.1 (U = 0 eV) and −1.9 eV (U = 5.0 eV) using the DFT+U method[33], while the GGA-PBE value of −4.18 eV is in good agreement with the experimental measurements (−3.57 to −4.03 eV). On the other hand, the *U*-value is usually chosen based on its accuracy in reproducing the electronic structures (i.e., experimental band gap) of the bulk materials. However, to simulate catalysts, it is better to choose *U* to fit the energy of the oxidation-reduction, since catalytic processes are controlled by energy difference[34]. The specific case in this work, namely CO_2RR on SAC surfaces, involves complex surface–adsorbate interactions, under which bulk-property derived *U* values in a locally changing surface environment may not adequately describe the reaction energetics[35,36].

Note that the results based on the GGA-PBE (the method used in this work) showed very good performance in understanding the reaction mechanisms and activity trends observed in experiments[37,38]. To verify the accuracy of the PBE results, we also investigated the CO_2RR pathways on the Pd@In_2Se_3 with the PBE+U method in which the previously validated *U* value of 8.00 eV was employed for the Pd 4*d* orbital[42]. Although the PBE+U results of limiting potentials are slightly (less than 0.2 V) larger than the PBE ones, the computed theoretical final product, reaction path, potential-liming step as well as the variation of limiting potential caused by polarization conversion are the same (see **Figure S25**). Thus, the standard PBE calculations provided accurate predictions to describe the CO_2RR activity.

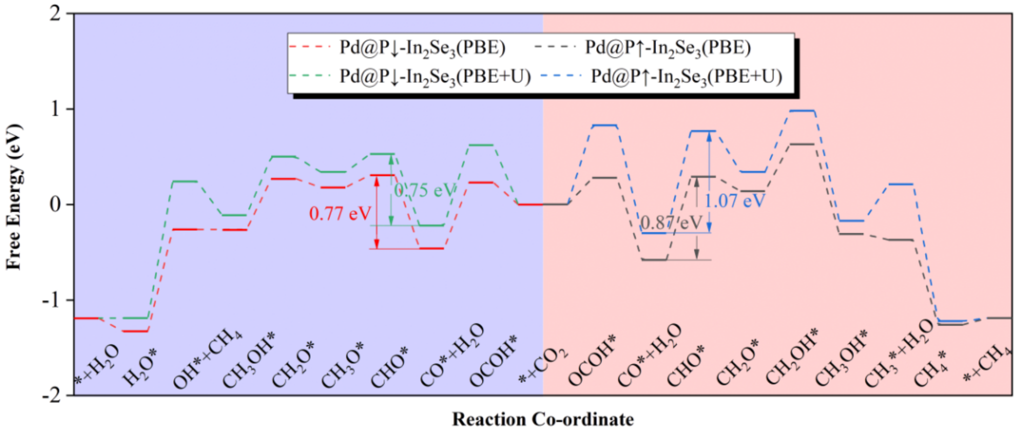

Figure S25 Comparison of the CO_2RR pathways on Pd@In_2Se_3 by using the PBE and PBE+U methods.

PBE *vs.* RPBE

It has been reported that the RPBE functional suggested by B. Hammer *et al.* could improve the chemisorption energetics of atoms and molecules on transition-metal surfaces.[43] In the present work, to evaluate the accuracy of the PBE functional, we also investigated the CO_2RR pathways on the Pd@In_2Se_3 with the RPBE functional. As shown in the **Figure S26**, except for the slightly increased limiting potential (less than 0.11 V), RPBE gives the same results as PBE, including the final product, reaction path, potential-liming step, as well as the variation of limiting potential caused by polarization conversion. Therefore, the conclusions obtained with the PBE functional hold true when the more accurate RPBE functional is used.

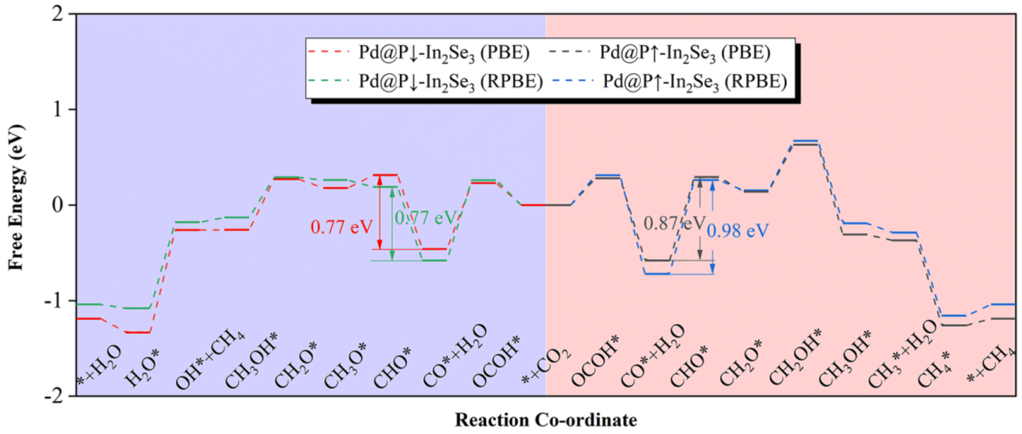

Figure S26 Comparison of CO_2RR pathways on Pd@In_2Se_3 by using the PBE and RPBE methods.

REFERENCES

1. Han G, Chen ZG, Drennan J, Zou J. Indium selenides: structural characteristics, synthesis and their thermoelectric performances. *Small* **10**, 2747-2765 (2014).

2. Ding W, *et al.* Prediction of intrinsic two-dimensional ferroelectrics in In2Se3 and other III_2-VI_3 van der Waals materials. *Nat. Commun.* **8**, 14956 (2017).

3. Popovic S, Tonejc A, Grzeta-Plenkovic B, Celustka B, Trojko R. Revised and new crystal data for indium selenides. *J. Appl. Crystallogr.* **12**, 416-420 (1979).

4. Osamura K, Murakami Y, Tomiie Y. Crystal Structures of α-and β-Indium Selenide, In_2Se_3. *J. Phys. Soc. Jpn.* **21**, 1848-1848 (1966).

5. Lutz HD, Fischer M, Baldus HP, Blachnik R. Zur polymorphie des In_2Se_3. *J. Less Common Metals* **143**, 83-92 (1988).

6. Zhou J, *et al.* Controlled Synthesis of High-Quality Monolayered alpha-In_2Se_3 via Physical Vapor Deposition. *Nano Lett.* **15**, 6400-6405 (2015).

7. Xiao J, *et al.* Intrinsic two-dimensional ferroelectricity with dipole locking. *Phys. Rev. Lett.* **120**, 227601 (2018).

8. Si M, *et al.* A ferroelectric semiconductor field-effect transistor. *Nat. Electron.* **2**, 580-586 (2019).

9. Cui C, *et al.* Intercorrelated In-Plane and Out-of-Plane Ferroelectricity in Ultrathin Two-Dimensional Layered Semiconductor In_2Se_3. *Nano Lett.* **18**, 1253-1258 (2018).

10. Bandurin DA, *et al.* High electron mobility, quantum Hall effect and anomalous optical response in atomically thin InSe. *Nat. Nanotechnol.* **12**, 223-227 (2017).

11. Mudd GW, *et al.* Tuning the bandgap of exfoliated InSe nanosheets by quantum confinement. *Adv. Mater.* **25**, 5714-5718 (2013).

12. Greeley J, Nørskov JK. Electrochemical dissolution of surface alloys in acids: Thermodynamic trends from first-principles calculations. *Electrochim. Acta* **52**, 5829-5836 (2007).

13. Guo X, Gu J, Lin S, Zhang S, Chen Z, Huang S. Tackling the Activity and Selectivity Challenges of Electrocatalysts toward the Nitrogen Reduction Reaction via Atomically Dispersed Biatom Catalysts. *J. Am. Chem. Soc.* **142**, 5709-5721 (2020).

14. Guo X, Lin S, Gu J, Zhang S, Chen Z, Huang S. Simultaneously Achieving High Activity and Selectivity toward Two-Electron O_2 Electroreduction: The Power of Single-Atom Catalysts. *ACS Catal.* **9**, 11042-11054 (2019).

15. WebBook NC. <07838353.pdf>. https://webbooknistgov/chemistry/.

16. Yan H, *et al.* Single-Atom Pd(1)/Graphene Catalyst Achieved by Atomic Layer Deposition: Remarkable Performance in Selective Hydrogenation of 1,3-Butadiene. *J. Am. Chem. Soc.* **137**, 10484-10487 (2015).

17. Zhang X, *et al.* Catalytically active single-atom niobium in graphitic layers. *Nat. Commun.* **4**, 1924 (2013).

18. Xiong Y, *et al.* Single-atom Rh/N-doped carbon electrocatalyst for formic acid oxidation. *Nat. Nanotechnol.* **15**, 390-397 (2020).

19. Zhao S, *et al.* One-Pot Pyrolysis Method to Fabricate Carbon Nanotube Supported Ni Single-Atom Catalysts with Ultrahigh Loading. *ACS Appl. Energ. Mater.* **1**, 5286-5297 (2018).

20. Chen M, *et al.* Stability of transition metals on Mg(0001) surfaces and their effects on hydrogen adsorption. *Int. J. Hydrogen Energ.* **37**, 309-317 (2012).

21. Alavi A, Hu P, Deutsch T, Silvestrelli PL, Hutter J. CO Oxidation on Pt(111): An Ab Initio Density Functional Theory Study. *Phys. Rev. Lett.* **80**, 3650-3653 (1998).

22. Hu T, Su H, Li Q, Kan E. Tunable ferroelectric single-atom catalysis of CO oxidation using a Pt/In_2Se_3 monolayer. *J. Mater. Chem. A* **8**, 20725-20731 (2020).

23. Tan X, Li XL, Yang GW. Theoretical strategy for self-assembly of quantum rings. *Phys. Rev. B* **77**, 245322 (2008).

24. Tan X, Ouyang G, Yang GW. Ordering Fe nanowire on stepped Cu (111) surface. *Appl. Phys. Lett.* **88**, 263116 (2006).

25. Tan X, Ouyang G, Yang GW. Surface smoothing of amorphous silicon thin films: Kinetic Monte Carlo simulations. *Phys. Rev. B* **73**, 195322 (2006).

26. Back S, Kim H, Jung Y. Selective Heterogeneous CO_2 Electroreduction to Methanol. *ACS Catal.* **5**, 965-971 (2015).

27. Kuhl KP, Cave ER, Abram DN, Jaramillo TF. New insights into the electrochemical reduction of carbon dioxide on metallic copper surfaces. *Energ. Environ. Sci.* **5**, 7050-7059 (2012).

28. Back S, Lim J, Kim NY, Kim YH, Jung Y. Single-atom catalysts for CO_2 electroreduction with significant activity and selectivity improvements. *Chem. Sci.* **8**, 1090-1096 (2017).

29. Kas R, Kortlever R, Milbrat A, Koper MTM, Mul G, Baltrusaitis J. Electrochemical CO_2 reduction on Cu_2O-derived copper nanoparticles: controlling the catalytic selectivity of hydrocarbons. *Phys. Chem. Chem. Phys.* **16**, 12194-12201 (2014).

30. Kim SK, Zhang Y-J, Bergstrom H, Michalsky R, Peterson A. Understanding the Low-Overpotential Production of CH_4 from CO_2 on Mo_2C Catalysts. *ACS Catal.* **6**, 2003-2013 (2016).

31. Wannakao S, Artrith N, Limtrakul J, Kolpak AM. Engineering Transition-Metal-Coated Tungsten Carbides for Efficient and Selective Electrochemical Reduction of CO_2 to Methane. *ChemSusChem* **8**, 2745-2751 (2015).

32. Torelli DA*, et al.* Nickel–Gallium-Catalyzed Electrochemical Reduction of CO2 to Highly Reduced Products at Low Overpotentials. *ACS Catal.* **6**, 2100-2104 (2016).

33. Shin H, Ha Y, Kim H. 2D Covalent Metals: A New Materials Domain of Electrochemical CO2 Conversion with Broken Scaling Relationship. *J. Phys. Chem. Lett.* **7**, 4124-4129 (2016).

34. Ma S*, et al.* Electroreduction of Carbon Dioxide to Hydrocarbons Using Bimetallic Cu–Pd Catalysts with Different Mixing Patterns. *J. Am. Chem. Soc.* **139**, 47-50 (2017).

35. Zhao Z, Lu G. Cu-Based Single-Atom Catalysts Boost Electroreduction of CO_2 to CH_3OH: First-Principles Predictions. *J. Phys. Chem. C* **123**, 4380-4387 (2019).

36. Wu J*, et al.* A metal-free electrocatalyst for carbon dioxide reduction to multi-carbon hydrocarbons and oxygenates. *Nat. Commun.* **7**, 13869 (2016).

37. Ma D, Zeng Z, Liu L, Huang X, Jia Y. Computational Evaluation of Electrocatalytic Nitrogen Reduction on TM Single-, Double-, and Triple-Atom Catalysts (TM = Mn, Fe, Co, Ni) Based on Graphdiyne Monolayers. *J. Phys. Chem. C* **123**, 19066-19076 (2019).

38. Cui X, An W, Liu X, Wang H, Men Y, Wang J. C_2N-graphene supported single-atom catalysts for CO_2 electrochemical reduction reaction: mechanistic insight and catalyst screening. *Nanoscale* **10**, 15262-15272 (2018).

39. Nørskov JK, Studt F, Abild-Pedersen F, Bligaard T. *Fundamental concepts in heterogeneous catalysis*. John Wiley & Sons (2014).

40. Nørskov JK*, et al.* Origin of the Overpotential for Oxygen Reduction at a Fuel-Cell Cathode. *J. Phys. Chem. B* **108**, 17886-17892 (2004).

41. Peterson AA, Abild-Pedersen F, Studt F, Rossmeisl J, Nørskov JK. How copper catalyzes the electroreduction of carbon dioxide into hydrocarbon fuels. *Energ. Environ. Sci.* **3**, 1311-1315 (2010).

42. Bennett JW, Grinberg I, Davies PK, Rappe AM. Pb-free semiconductor ferroelectrics: A theoretical study of Pd-substitutedBa($Ti_{1-x}Ce_x$)O_3solid solutions. *Phys. Rev. B* **82**, 184106 (2010).

43. Hammer B, Hansen LB, Nørskov JK. Improved adsorption energetics within density-functional theory using revised Perdew-Burke-Ernzerhof functionals. *Phys. Rev. B* **59**, 7413-7421 (1999).
